# Genetic Predisposition to Coronary Artery Disease in Type 2 Diabetes Mellitus

**DOI:** 10.1161/CIRCGEN.119.002769

**Published:** 2020-08-13

**Authors:** Natalie R. van Zuydam, Claes Ladenvall, Benjamin F. Voight, Rona J. Strawbridge, Juan Fernandez-Tajes, N. William Rayner, Neil R. Robertson, Anubha Mahajan, Efthymia Vlachopoulou, Anuj Goel, Marcus E. Kleber, Christopher P. Nelson, Lydia Coulter Kwee, Tõnu Esko, Evelin Mihailov, Reedik Mägi, Lili Milani, Krista Fischer, Stavroula Kanoni, Jitender Kumar, Ci Song, Jaana A. Hartiala, Nancy L. Pedersen, Markus Perola, Christian Gieger, Annette Peters, Liming Qu, Sara M. Willems, Alex S.F. Doney, Andrew D. Morris, Yan Zheng, Giorgio Sesti, Frank B. Hu, Lu Qi, Markku Laakso, Unnur Thorsteinsdottir, Harald Grallert, Cornelia van Duijn, Muredach P. Reilly, Erik Ingelsson, Panos Deloukas, Sek Kathiresan, Andres Metspalu, Svati H. Shah, Juha Sinisalo, Veikko Salomaa, Anders Hamsten, Nilesh J. Samani, Winfried März, Stanley L. Hazen, Hugh Watkins, Danish Saleheen, Andrew P. Morris, Helen M. Colhoun, Leif Groop, Mark I. McCarthy, Colin N.A. Palmer

**Affiliations:** 1 Pat Macpherson Center for Pharmacogenetics & Pharmacogenomics, Cardiovascular & Diabetes Medicine (N.R.v.Z., C.N.A.P.), School of Medicine, University of Dundee.; 2 Division of Molecular & Clinical Medicine (A.S.F.D.), School of Medicine, University of Dundee.; 3 Oxford Center for Diabetes, Endocrinology & Metabolism, Radcliffe Department of Medicine (N.R.v.Z., N.W.R., N.R.R., A. Mahajan, M.I.Mc), University of Oxford, United Kingdom.; 4 Wellcome Center for Human Genetics (N.R.v.Z., J.F.T., N.W.R., N.R.R, A. Mahajan, A.G., H.W., A.P.M., M.I.Mc), University of Oxford, United Kingdom.; 5 Division of Cardiovascular Medicine (A.G., H.W.), University of Oxford, United Kingdom.; 6 Department of Clinical Sciences, Diabetes & Endocrinology, Lund University Diabetes Center, Malmö, Sweden (C.L., L.G.).; 7 Department of Systems Pharmacology & Translational Therapeutics (B.F.V.); 8 Department of Genetics (B.F.V.); 9 Institute for Translational Medicine & Therapeutics (B.F.V.); 10 Cardiovascular Institute, University of Pennsylvania, Perelman School of Medicine, Philadelphia, PA (L.Q., M.P.R.).; 11 Cardiovascular Medicine Unit, Department of Medicine Solna, Center for Molecular Medicine, Karolinska Institutet, Karolinska University Hospital Solna, Stockholm, Sweden (R.J.S., A.H.).; 12 Department of Human Genetics, Wellcome Trust Sanger Institute, Hinxton, United Kingdom (N.W.R.).; 13 Transplantation Laboratory, Haartman Institute (E.V.), University of Helsinki, Helsinki, Finland.; 14 Research Program for Clinical & Molecular Metabolism, Faculty of Medicine (M.P.), University of Helsinki, Helsinki, Finland. Vth Department of Medicine (Nephrology, Hypertensiology, Rheumatology, Endocrinology, Diabetology), Medical Faculty Mannheim, Heidelberg University, Mannheim, Germany.; 15 Department of Cardiovascular Sciences, University of Leicester (C.P.N., N.J.S.).; 16 NIHR Leicester Biomedical Research Center, Glenfield Hospital, Leicester, United Kingdom (C.P.N., N.J.S.).; 17 Division of Cardiology, Department of Medicine, Duke University Medical Center (S.H.S.); 18 Duke Molecular Physiology Institute, Duke University, Durham, NC (L.C.K., S.H.S.).; 19 Estonian Genome Center (T.E., E.M., R.M., L.M., K.F., M.P., A. Metspalu), University of Tartu, Tartu, Estonia.; 20 Institute of Cell & Molecular Biology (A. Metspalu), University of Tartu, Tartu, Estonia.; 21 Center for Genomic Health (S.K.), Queen Mary University of London, London, United Kingdom.; 22 William Harvey Research Institute, Barts & the London Medical School (S.K., P.D.), Queen Mary University of London, London, United Kingdom.; 23 Department of Medical Sciences, Molecular Epidemiology & Science for Life Laboratory (J.K., C.S., E.I.); 24 Department of Immunology, Genetics and Pathology, Medical Genetics & Genomics, Uppsala University, Uppsala, Sweden (C.S.).; 25 Center for Computational Biology & Bioinformatics, Amity Institute of Biotechnology, Amity University Uttar Pradesh, Noida, India (J.K.).; 26 Framingham Heart Study (C.S.).; 27 Population Sciences Branch, National Heart, Lung & Blood Institute, National Institute of Health, Framingham, MA (C.S.).; 28 Department of Preventive Medicine, Keck School of Medicine, University of Southern California, Los Angeles, CA (J.A.H.).; 29 Department of Medical Epidemiology & Biostatistics, Karolinska Institutet, Stockholm, Sweden (N.L.P.).; 30 National Institute for Health and Welfare, Helsinki, Finland (M.P., V.S.).; 31 German Center for Diabetes Research (DZD), München-Neuherberg (C.G., A.P., H.G.).; 32 Clinical Cooperation Group Type 2 Diabetes (C.G., H.G.), Helmholtz Zentrum München, Neuherberg, Germany.; 33 German Research Center for Environmental Health & Institute of Genetic Epidemiology (C.G., A.P.), Helmholtz Zentrum München, Neuherberg, Germany.; 34 Research Unit of Molecular Epidemiology, Institute of Epidemiology (H.G.), Helmholtz Zentrum München, Neuherberg, Germany.; 35 Clinical Cooperation Group Nutrigenomics & Type 2 Diabetes (H.G.), Helmholtz Zentrum München, Neuherberg, Germany.; 36 DZHK (German Center for Cardiovascular Research), partner site Munich Heart Alliance, Munich, Germany (A.P.).; 37 Department of Epidemiology, Erasmus Medical Center, Rotterdam, the Netherlands (S.M.W., C.v.D.).; 38 The Usher Institute of Population Health Sciences & Informatics (A.D.M.), University of Edinburgh, Edinburgh, U.K.; 39 MRC Institute of Genetics & Molecular Medicine (H.M.C.), University of Edinburgh, Edinburgh, U.K.; 40 Health Data Research UK, London, United Kingdom (A.D.M.).; 41 Department of Nutrition (Y.Z., F.B.H., L.Q.); 42 Department of Epidemiology, Harvard School of Public Health, Boston, MA (F.B.H.).; 43 Ministry of Education Key Laboratory of Contemporary Anthropology, School of Life Sciences, Fudan University, Shanghai, China (Y.Z.).; 44 University “Magna Graecia” of Catanzaro, Italy (G.S.).; 45 Channing Division of Network Medicine, Department of Medicine, Brigham and Women’s Hospital & Harvard Medical School, Boston, MA (F.B.H.).; 46 Department of Epidemiology, School of Public Health & Tropical Medicine, Tulane University, New Orleans, LA (L.Q.).; 47 Faculty of Health Sciences, Institute of Clinical Medicine, Internal Medicine, University of Eastern Finland (M.L.).; 48 Kuopio University Hospital, Finland (M.L.).; 49 Faculty of Medicine, University of Iceland. deCODE Genetics, Reykjavik, Iceland (U.T.).; 50 Division of Cardiovascular Medicine, Department of Medicine, Stanford University School of Medicine (E.I.).; 51 Stanford Cardiovascular Institute (E.I.); 52 Stanford Diabetes Research Center, Stanford University, Stanford, CA (E.I.).; 53 Princess Al-Jawhara Al-Brahim Center of Excellence in Research of Hereditary Disorders (PACER-HD), King Abdulaziz University, Jeddah, Saudi Arabia (P.D.).; 54 Broad Institute of MIT & Harvard, Cambridge (S.K.).; 55 Cardiology Division, Center for Human Genetic Research (S.K.), Massachusetts General Hospital & Harvard Medical School, Boston, MA.; 56 Cardiovascular Research Center (S.K.), Massachusetts General Hospital & Harvard Medical School, Boston, MA.; 57 Heart & Lung Center, Helsinki University Hospital (J.S.) and Institute for Molecular Medicine Finland (FIMM), Helsinki University, Helsinki, Finland.; 58 Synlab Academy, Synlab Holding Deutschland GmbH, Mannheim, Germany (W.M.).; 59 Clinical Institute of Medical & Chemical Laboratory Diagnostics, Medical University of Graz, Austria (W.M.).; 60 Lerner Research Institute, Heart & Vascular Institute, Cleveland Clinic, Cleveland, OH (S.L.H.).; 61 Department of Biostatistics & Epidemiology, University of Pennsylvania, Philadelphia, PA (D.S.).; 62 Center for Non-Communicable Diseases, Karachi, Pakistan (D.S.).; 63 Department of Biostatistics, University of Liverpool, Liverpool, U.K. (A.P.M.).; 64 Division of Musculoskeletal & Dermatological Sciences, University of Manchester, Manchester, U.K. (A.P.M.).; 65 Public Health, NHS Fife, Kirkcaldy, Fife, U.K. (H.M.C.).; 66 Oxford NIHR Biomedical Research Center, Oxford University Hospitals NHS Foundation Trust, John Radcliffe Hospital, Oxford, United Kingdom (M.I.Mc).

**Keywords:** blood pressure, coronary artery disease, diabetes mellitus, genome-wide association study, risk factors

## Abstract

Coronary artery disease (CAD) is accelerated in subjects with type 2 diabetes mellitus (T2D).

**Methods:**

To test whether this reflects differential genetic influences on CAD risk in subjects with T2D, we performed a systematic assessment of genetic overlap between CAD and T2D in 66 643 subjects (27 708 with CAD and 24 259 with T2D). Variants showing apparent association with CAD in stratified analyses or evidence of interaction were evaluated in a further 117 787 subjects (16 694 with CAD and 11 537 with T2D).

**Results:**

None of the previously characterized CAD loci was found to have specific effects on CAD in T2D individuals, and a genome-wide interaction analysis found no new variants for CAD that could be considered T2D specific. When we considered the overall genetic correlations between CAD and its risk factors, we found no substantial differences in these relationships by T2D background.

**Conclusions:**

This study found no evidence that the genetic architecture of CAD differs in those with T2D compared with those without T2D.

## Abstract 2

Supplemental Digital Content is available in the text.

There is considerable variation in the presentation, severity, and pathology of coronary artery disease (CAD) between subjects with type 2 diabetes mellitus (T2D) and those with no history of diabetes mellitus. Subjects with T2D have more extensive and severe atherosclerosis, suffer more silent infarcts, and are more prone to thrombosis than subjects without diabetes mellitus.^1–3^ The mechanisms by which T2D accelerates CAD are poorly understood. In principle, the acceleration of CAD in T2D may be attributed to features that jointly predispose subjects to T2D and CAD or to factors intrinsic to the T2D state that increase the risk of CAD, such as hyperglycemia, insulin resistance, and chronic inflammation.^4^

Predisposition to both CAD and T2D has a substantial genetic component (with ≈163 CAD risk and ≈403 for T2D association signals identified to date in subjects of European Ancestry)^5,6^ and Mendelian randomization studies support a causal role for T2D in the development of CAD.^7–9^ A Mendelian randomization study found that the average CAD risk per T2D allele was lower than expected (for the 44 T2D associated variants assessed) compared with the increased risk of CAD attributed to T2D by epidemiological studies.^7^ This indicated that the T2D associated variants did not account for all the risk of CAD observed in subjects with T2D. Few variants have been associated with both CAD and T2D: a variant near *IRS1* was associated with both diseases at genome-wide significance (*P*≤5×10^-8^) and 8 other loci at a lower significance level.^9^ Given that there are few variants jointly associated with CAD and T2D, it is unsurprising that there is sparse evidence for overlapping pathways contributing to both diseases.^10^

A recent study conducted in the UK Biobank found no evidence of differential effects of CAD risk variants by T2D status. However, in this study, the sample size was relatively small (3968 CAD cases and 11 698 controls).^11^ Another study found that a genetic risk score constructed from known CAD loci was associated with CAD in subjects with T2D, indicating that variants identified in the general population were predictive of CAD in the context of T2D.^12^ What has not been systematically addressed in a large sample is whether there is a quantitative or qualitative difference in the pattern of loci influencing risk of CAD among subjects with T2D when compared with those without the condition.

We conducted a comprehensive investigation of genetic differences in the determinants of CAD between subjects with and without T2D in a large sample. The discovery meta-analysis included 66 643 subjects (of whom 27 708 had CAD and 24 259 had T2D), and we sought replication for a subset of variants in a further 117 787 samples (16 694 with CAD; 11 537 subjects with T2D).

## Methods

**Figure 1.**
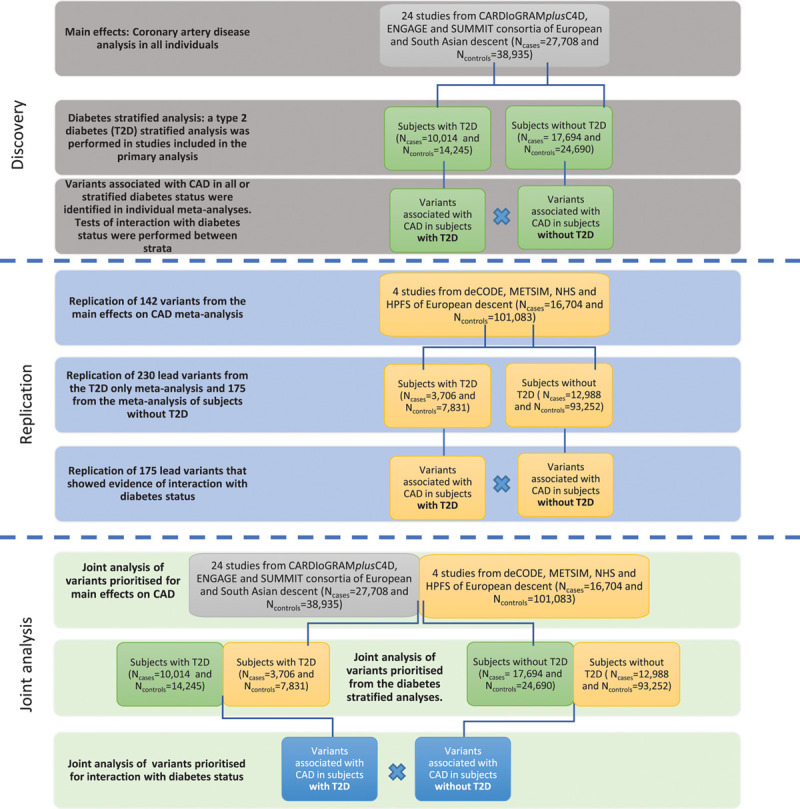
**Study design.** In the discovery meta-analyses, we performed 4 different meta-analyses of coronary artery disease (CAD): in all individuals irrespective of Type 2 diabetes mellitus (T2D) status; in all individuals corrected for T2D stats; and stratified by T2D status. We examined allelic effects within strata to identify stratum-specific CAD associated variants, and between strata to identify variants that may interact with T2D status to modify the risk of CAD. We selected variants that achieved *P*<1×10^-4^ for association with CAD in at least one of the following analyses: all individuals combined regardless of T2D status; subjects with T2D only; subjects without diabetes mellitus; or the interaction analysis. The replication analysis was performed in independent samples using the same study design as the discovery analysis. CARDIoGRAMplusC4D indicates Coronary Artery Disease Genome Wide Replication and Meta-Analysis (CARDIoGRAM) Plus the Coronary Artery Disease (C4D) Genetics; ENGAGE, European Network for Genetic and Genomic Epidemiology; HPFS, Health Professionals Follow-Up Study; METSIM, The Metabolic Syndrome in Men Study; NHS, Nurses’ Health Study; and SUMMIT, Surrogate Markers for Micro- and Macro-Vascular Hard End Points for Innovative Diabetes Tools.

An overview of the study design is illustrated in Figure 1 and the methods are provided in the Data Supplement. The summary statistics have been made available via figshare (10.6084/m9.figshare.7811639). This study made use of data generated from individual studies for which the relevant institutional review board approval had been obtained and all participants consented to inclusion in individual studies.

## Results

### Identification of CAD Cases, CAD Controls, and Subjects With Diabetes Mellitus

This study was performed using full summary statistics from CAD case-control analyses performed separately in subjects with T2D and subjects with no history of diabetes mellitus. The discovery meta-analyses included 27 708 CAD cases (of whom 10 014 had T2D) and 38 935 subjects with no history of CAD (14 245 with T2D) from 23 studies of European descent and one study of South Asian descent, assembled from the CARDIoGRAMplusC4D (Coronary Artery Disease Genome Wide Replication and Meta-Analysis (CARDIoGRAM) Plus the Coronary Artery Disease (C4D) Genetics), ENGAGE (European Network for Genetic and Genomic Epidemiology), and SUMMIT (Surrogate Markers for Micro- and Macro-Vascular Hard End Points for Innovative Diabetes Tools) consortia (Figure 1 and Tables I and II in the Data Supplement). Replication of selected signals was sought in an independent sample of 16 694 CAD cases (3706 with T2D) and 101 093 controls with no history of CAD (7831 with T2D) from 4 studies of European descent with existing genome-wide association study from deCODE, the NHS (Nurses’ Health Study), the METSIM study (The Metabolic Syndrome in Men), and the HPFS (Health Professionals Follow-Up Study; Tables III and IV in the Data Supplement). None of the studies contained overlapping samples.

### Main Effects of Variants on CAD

We first set out to identify variants that were associated with CAD in the complete sample set. We performed 2 meta-analyses, the first compared CAD cases to controls without reference to T2D status, whereas the second repeated the analysis adjusted for T2D status. In both analyses, we confirmed many of the previously reported CAD associated loci at genome-wide significance (*P*≤5×10^-8^), including *SORT1/CELSR2*, *WDR12, PHACTR1, TCF21*, 9p21.3, *CXCL12*, and *ADAMTS7*. We selected 142 variants that achieved *P*≤5×10^-4^ in either the unadjusted or the T2D- adjusted analyses for replication analyses.

**Figure 2.**
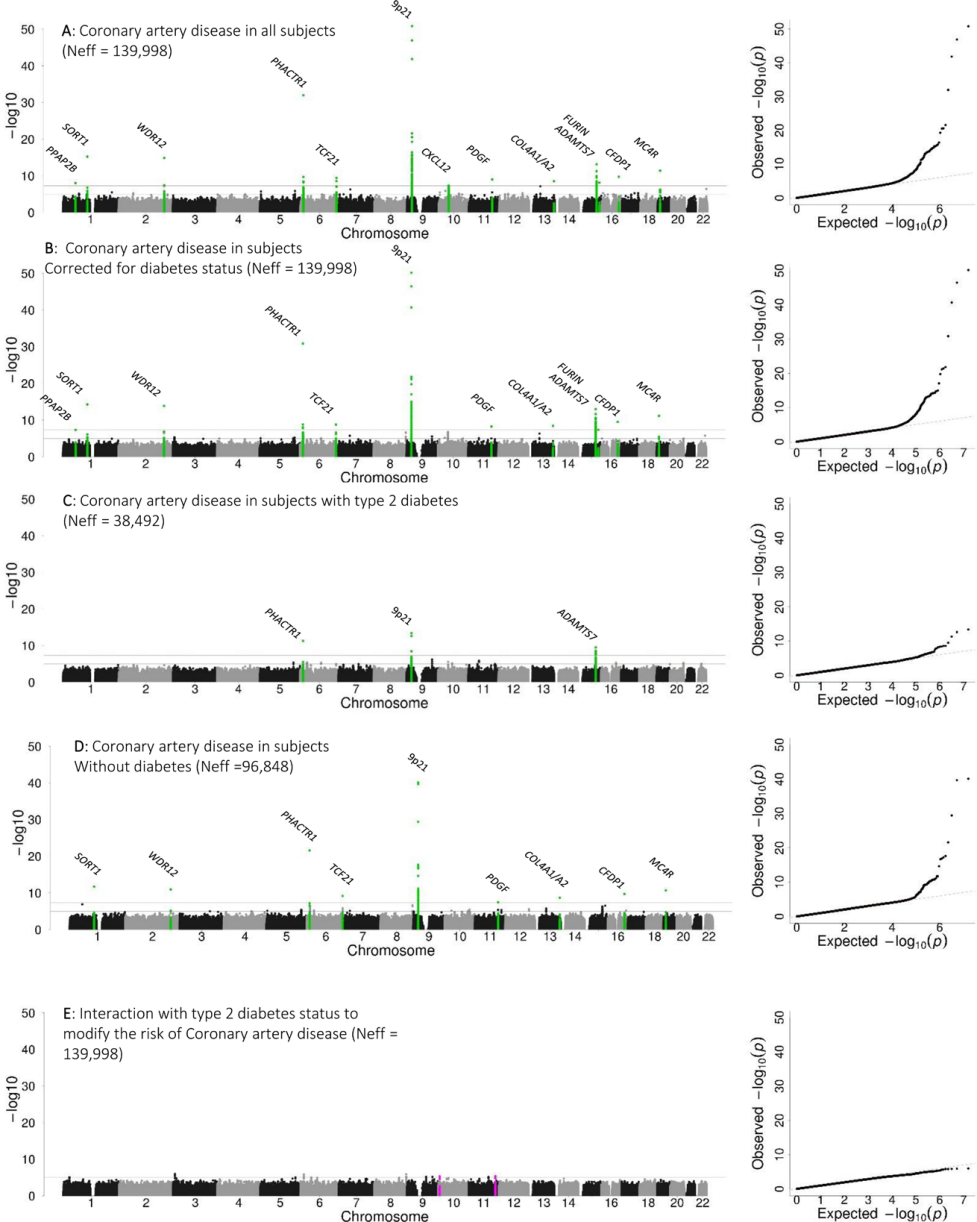
**Five genetic association study meta-analyses were performed to investigate the genetic architecture of coronary artery disease (CAD) in the context of Type 2 diabetes mellitus (T2D).** Manhattan and QQ plots from (**A**) a meta-analysis that combined allelic effects on CAD from subjects with T2D status and without diabetes mellitus and (**B**) corrected for T2D status to identify variants associated with CAD irrespective of T2D status; (**C**) a meta-analysis of allelic effects on CAD in subjects with T2D to identify loci that may influence the development of CAD in the context of T2D; (**D**) a meta-analysis of allelic effects on CAD in the absence of diabetes mellitus to identify loci that may influence the development of CAD in the absence of diabetes mellitus; and (**E**) an interaction analysis to identify loci that may interact with T2D to modify the risk of CAD. The effective sample size was based on the combined discovery and replication sample of 184 250 subjects.

We had access to full summary statistics for the discovery analysis but not from the replication cohorts. We requested summary statistics for selected variants from replication cohorts. Thus, we performed a joint analysis of the estimates from the discovery and replication analyses. In the joint analysis, we expanded the set of known CAD loci detected in this meta-analysis from 7 to 13 reaching genome-wide significance in our dataset (Figure 2A and 2B and Table V in the Data Supplement). For published CAD variants, the risk allele identified in this meta-analysis was the same as the published risk allele for variants associated with CAD *P*≤1×10^-3^ (Figure II and Table V in the Data Supplement).^5^ This reflects, in part, an overlap of samples included in these various analyses (Figure II and Table V in the Data Supplement).

### Stratified Analysis

The second approach we used to identify any loci at which CAD risk effects (*P*≤5×10^-8^) were influenced by the presence or absence of T2D, involved a T2D-stratified meta-analysis of CAD risk. In the discovery phase of this stratified analysis, 3 known CAD loci reached genome-wide significance: *ADAMTS7* in subjects with T2D and 9p21.3 and *PHACTR1* in the analysis of subjects without diabetes mellitus (Table V in the Data Supplement). The allelic effects and association signals at the previously reported CAD loci did not show any systematic difference according to T2D background (Figure I in the Data Supplement).

We selected 230 lead variants for replication from the T2D-only analysis and 175 lead variants from the analysis of subjects without diabetes mellitus for replication based on a stratum-specific CAD association of *P*≤1×10^-4^. In the joint analysis (discovery and replication), we found no novel CAD risk signals in either stratum (Figure 2C and 2D and Table V in the Data Supplement). Three loci were associated with CAD in subjects with T2D, and these overlapped loci associated with CAD in subjects without diabetes mellitus (Figure 2). The different number of loci associated with CAD by T2D background reflects a difference in power (ie, sample size) to detect associations rather than a systematic difference by T2D background.

### Interaction Analysis

In a complementary analysis to the stratified analysis, we performed a T2D interaction analysis (see Methods in the Data Supplement) to identify variants that interacted with T2D status to modify the risk of CAD. We calculated the interaction *P* values based on summary statistics from the T2D stratified analyses of CAD and not from a meta-analysis of interaction terms. We adopted this approach to maximize the number of samples used to estimate interactive effects (see Methods in the Data Supplement). The interaction analysis was performed by comparing the allelic effects (on the log-odds scale) on CAD risk for each variant between T2D strata. The allelic effects and their associated standard errors for CAD risk estimated in T2D stratified meta-analyses were compared using GWAMA v2.1.^13^ The smaller the *P*_*interaction*_ the larger difference in allelic effects on CAD risk by T2D status.

The top interaction in the discovery analysis was represented by rs712755, near *GRM7* (*P*_*interaction*_=4.6×10^-7^). This variant had opposing effects on CAD risk dependent on T2D context (effect allele frequency, 0.71, odds ratio [OR]_T2D_, 0.82 [0.74–0.90], OR_Nodiabetesmellitus_, 1.14 [1.06–1.23]).

We sought replication for 175 loci, including *GRM7*, with at least modest evidence of interaction with T2D status (*P*_*interaction*_ ≤1×10^-4^). We performed a joint interaction meta-analysis of the discovery and replication data and defined replication as a combined (discovery+replication) *P*_*interaction*_<2.9×10^-4^ (0.05/175; that corrects for the number of loci selected for replication) and a joint *P*_*interaction*_< discovery *P*_*interaction*_. The latter indicates directionally consistent allelic effects by T2D stratum in the discovery and replication stages.

The interaction at *GRM7*, represented by rs712755, did not replicate (replication *P*_*interaction*_=0.36) and none of the other 174 loci met the criteria for replication. Overall, there was no evidence for loci that interacted with T2D status to modify the risk of CAD based on this interaction analysis.

We also examined the known CAD loci for evidence of interaction. Of the 163 known variants for CAD, 161 were present in our data. We applied a Bonferroni correction of *P*_*interaction*_≤3.1×10^-4^ (0.05/161; correcting for the number of known CAD loci). None of the established CAD loci interacted with T2D status to modify the risk of CAD (Table V in the Data Supplement). A variant located near *GLUL* (rs10911021) had been associated with CAD in subjects with T2D.^14^ In the current study, rs10911021 showed no association with CAD in subjects with T2D (*P*=0.54) and had no evidence of interaction with T2D status (*P*_*interaction*_=0.46; Figure III in the Data Supplement).

### Power to Detect Interactions

A substantial challenge in detecting loci that interact with T2D to modify the risk of CAD is sufficient sample size. Even in this large discovery sample of 66 643 subjects (27 708 with CAD), we had <80% power to detect interactions with at least a 20% difference in allelic odds between strata (ie, OR_Nodiabetesmellitus_, 1.00 versus OR_T2D_, 1.20) for risk allele frequency>10% at α=1×10^-4^ (the threshold for replication selection in the interaction analysis; see Methods in the Data Supplement). This was only for interactions where there were opposite allelic effects in strata or where there was a null allelic effect on CAD in one stratum (ie, OR_Nodiabetesmellitus_, 1.00) and a large (ie, OR_T2D_, 1.20) allelic effect on CAD in the other stratum (Figure IIA and IIB in the Data Supplement). We had little power to detect interactions where allelic effects on CAD were in the same direction in both strata (see Methods and Figure IIC in the Data Supplement). In the replication sample of 117 787 samples (16 694 with CAD) at an α=0.05, we observed similar patterns of power to detect associations with opposing effects by stratum. Thus, we would be unlikely (in this sample size) be able to detect smaller interaction effects or those involving rare alleles.

### Genetic Overlap With Risk Factors

We have comprehensively interrogated variants for association with CAD in the context of T2D but not risk factors of both T2D and CAD. There may be a different effect of these risk factors on CAD by T2D context, which may explain some of the increased risk of CAD in subjects with T2D. First, we performed genetic correlation analyses using LDHub (a centralised database of summary-level GWAS results and a web interface for LD score regression) to estimate the overall genetic correlation (based on all variants) between risk factors and CAD separately by T2D background. ^15^ Subsequently, a heterogeneity test was performed on the risk factor genetic correlation estimates with CAD by T2D background to identify risk factors that may have a variable correlation with CAD based on T2D background. Overall, we found no difference in the genetic correlation between 106 risk factors and CAD by T2D status (Figure IV and Table VI in the Data Supplement).

To investigate this further but only in a subset of variants associated with risk factors at genome-wide significance (*P*≤5×10^−8^), we constructed weighted genetic risk scores for seventeen traits related to obesity,^16–18^ hypertension,^19^ lipids,^20^ diabetes mellitus,^6,21^ glycaemic traits, and insulin resistance.^22–28^ These genetic risk scores included between 10 and 403 single nucleotide polymorphisms for each phenotype. We tested these for CAD association in the T2D unadjusted (main) analysis, as well as in the T2D-stratified analyses, where we performed a test for heterogeneity for different effects on CAD by T2D background (Methods in the Data Supplement). We adopted a significance threshold of *P*≤2.9×10^-3^ that accounted for the 17 genetic risk scores, but not for the multiple CAD associations performed. Genetic risk scores for LDL-C (low-density lipoprotein cholesterol), body mass index, and systolic blood pressure were associated with CAD irrespective of T2D background (Figure V and Table VII in the Data Supplement). Collectively, these analyses provide no evidence to support T2D-stratified differences in CAD risk as conveyed by variants influencing phenotypes known to contribute to CAD development.

## Discussion

There is a well-established causal role for T2D in increased risk of CAD. However, this increased risk could not be explained by differences in genetic architecture of CAD between individuals with and without diabetes mellitus. We found no difference in the effects of known CAD loci on the risk of CAD by T2D status. We also found no variants of large effect specifically associated with CAD in the context of T2D. We also found no differences in the effects of risk factors on CAD by T2D background based on analyses that used the genetic variation contributing to these risk factors. Indicating that the genetic variants associated with these risk factors do not have a differential effect on CAD risk by T2D background.

There are many factors that will influence the power to detective genuine interactive effects. Identification of interactive effects requires a large sample size particularly when conducting a genome-wide interaction analysis.^29^ Even in this study that included 66 643 subjects (considerably larger than previous efforts), we were underpowered to identify variants with small differences in effect on CAD risk by T2D status. If interaction effects do exist, these effects are likely to be modest and only detectable in a much larger sample size.

The accuracy of the phenotype definition will also affect the power to detect interactive effects. Diagnosis of T2D is often contemporaneous to CAD diagnosis and may not reflect the actual onset of diabetes mellitus. We are uncertain of the stage of T2D development when risk of CAD begins to increase. There is evidence of increased vascular risk before the onset of clinically diagnosed T2D.^30^ Taking this variability into account, we defined CAD cases with T2D as those that had a diagnosis of T2D up to 5 years after a CAD event with no minimum duration of diabetes mellitus. This also allowed us to increase the sample size by including cross-sectional studies for which information on the duration of diabetes mellitus may not be available. We were unable to account for the attenuation of genetic effects due to the misclassification of subjects who may develop CAD and or T2D outside of the study observation period.

This study shows that difference in risk of CAD between subjects with and without T2D cannot be explained by variants of large effect or differences in the genetic variation contributing to known risk factors of either T2D or CAD. There are several other mechanisms, outside the scope of the current study, that could explain some of the increased risk of CAD in subjects with T2D. There could be epigenetic changes induced by some feature of the T2D state. For example, hyperglycemia has been shown to cause epigenetic changes altering gene expression in vascular cells leading to endothelial dysfunction, a hallmark of atherosclerosis.^31^ Although the evidence for overlapping pathways between CAD and T2D is sparse, treatment of one disease can increase the risk of the other. Statins known to reduce the risk of CAD have been shown to increase the risk of T2D, whereas some thialidazones, used to treat insulin resistance in subjects with T2D, increase the risk of CAD.^32^ It is likely that the T2D state perturbs or exacerbates some common atherosclerotic processes rather than through T2D background specific genes/pathways to increase the risk of CAD in subjects with T2D.

## Sources of Funding

This work was supported by the European Union Framework Programme 7 (FP7/2007-2013) for the Innovative Medicine Initiative (IMI) under grant agreement n° IMI/115006 (the SUMMIT [Surrogate Markers for Micro- and Macro-Vascular Hard End Points for Innovative Diabetes Tools] consortium); Aarno Koskelo Foundation; Academy of Finland (no. 263401; no. 2676882); American Heart Association (13SDG14330006); AstraZeneca; AtheroSysMed (Systems medicine of coronary heart disease and stroke); British Heart Foundation Centre of Research Excellence at Oxford; ERC269045-Gene Target T2D grant; Estonian Research Council (IUT20-60, PUT1660 and PUT1665P); Estonian Center of Genomics/Roadmap II (project No. 2014-2020.4.01.16-0125); European Union (no. 692145; no. 633589; no. 313010; LSHM-CT-2007-037273; no. 201668; 2014-2020.4.01.15-0012;QLG1-CT-2002-00896; EU/QLRT-2001-01254; QLG2-CT-2002-01254 HEALTH-F2-2013-601456); Finnish Foundation for Cardiovascular research; Gentransmed - Centre of Excellence for Genomics and Translational Medicine; German Ministry of Education and Research (no. 01ZX1313A-K); Helsinki University Central Hospital special government funds (TYH7215, TKK2012005, TYH2012209, TYH2014312); Juvenile Diabetes Research Foundation (JDRF, 2-SRA-2014-276-Q-R); National Institute of Diabetes and Digestive and Kidney diseases (NIDDK, 5R01DK106236; U01-DK066134; U01-DK105535; R01DK101478); National Heart, Lung and Blood Institute (NLHBI, R01HL103866); National Institute for Health Research (NIHR); Personalized diagnostics and treatment of high risk coronary artery disease patients (RiskyCAD; 305739); Sigrid Juselius Foundation; Finnish Academy (no. 269517); Finnish Foundation for Cardiovascular Research; Foundation for Strategic Research and Stockholm County Council (560283; 592229); Juho Vainio Foundation; Knut and Alice Wallenberg Foundation; Ministry for Higher Education; Strategic Cardiovascular and Diabetes Programmes of Karolinska Institutet and Stockholm County Council; Swedish Foundation for Strategic Research (SSF; ICA08-0047); Swedish Heart-Lung Foundation; Swedish Research Council (project 8691; 2015-02558; 2016-00598; M-2005-1112 and 2009-2298); Torsten and Ragnar Söderberg Foundation; W.W. Smith Charitable Trust (H1201); Wellcome Trust Institutional strategic support fund; Yrjö Jahnsson Foundation.

## Disclosures

Authors have disclosed possible conflicts of interest and have confirmed that these are unrelated to the work described in this article. Dr Ingelsson is a scientific advisor for Precision Wellness. Dr Salomaa has consulted for Novo Nordisk and Sanofi and has ongoing research collaboration with Bayer (all unrelated to the present study). Dr März reports employment with Synlab Holding Deutschland GmbH and has received grants or personal fees from Abbott Diagnostics; Aegerion Pharmaceuticals; AMGEN; AstraZeneca; BASF Pharma Solutions; Danone Research; MSD; Sanofi; Siemens Diagnostics; and Synageva. Dr Colhoun receives research support and honoraria from and is also a member of the advisory panels and speaker’s bureaus for Sanofi Aventis, Regeneron, and Eli Lilly. Dr Colhoun has been a member of Data and Safety Monitoring Board of the Advisory Panel for the CANTOS Trial (Canakinumab. Anti-Inflammatory Thrombosis Outcome Study; Novartis Pharmaceuticals). Dr Colhoun also receives or has recently received nonbinding research support from Roche Pharmaceuticals, Pfizer, Inc, Boehringer Ingelheim, and AstraZeneca. Dr Colhoun is a shareholder of Roche Pharmaceuticals and Bayer. Dr McCarthy has served on advisory panels for Pfizer, NovoNordisk, and Zoe Global, has received honoraria from Merck, Pfizer, Novo Nordisk, and Eli Lilly, and research funding from Abbvie, AstraZeneca, Boehringer Ingelheim, Eli Lilly, Janssen, Merck, NovoNordisk, Pfizer, Roche, Sanofi Aventis, Servier, and Takeda. As of June 2019, Dr McCarthy is an employee of Genentech and a holder of Roche stock. As of September 2019, Dr van Zuydam is an employee of AstraZeneca. As of 2016, Dr Vlachopoulou is an employee of Medpace. The other authors report no conflicts.

## Supplementary Material



## Appendix

CARDIoGRAMplusC4D

John Danesh, Jeanette Erdmann, Dongfeng Gu, Jaspal S. Kooner, Robert Roberts, Heribert Schunkert, Themistocles L. Assimes, Stefan Blankenberg, Bernhard O. Boehm, John C. Chambers, Robert Clarke, Rory Collins, George Dedoussis, Paul W. Franks, G. Kees Hovingh, Bong-Jo Kim, Terho Lehtimäki, Ruth McPherson, Markku S Nieminen, Christopher O'Donnell, Samuli Ripatti, Manjinder S Sandhu, Stefan Schreiber, Agneta Siegbahn, Cristen J. Willer, Pierre A. Zalloua

SUMMIT

Michael Mark, Timo Kanninen, Barbara Thorand, Giuseppe Remuzzi, David Dunger, Angela Shore, Ulf Smith, Per-Henrik Groop, Seppo Ylä-Herttuala, Claudio Cobelli, Riccardo Bellazzi, Ele Ferrannini, Carlo Patrono, Pirjo Nuutila, Paul McKeague, Birgit Steckel-Hamann, Li-ming Gan, Everson Nogoceke, Piero Tortoli, Bernd Jablonka, Mary-Julia Brosnan
