# Supplementary material for: Genetic Predisposition to Coronary Artery Disease in Type 2 Diabetes Mellitus
Source: Circ Genom Precis Med. 2020 Aug 13;13(6):e002769. doi: 10.1161/CIRCGEN.119.002769 (PMC7748049; doi:10.1161/CIRCGEN.119.002769)
Supplement: Supplementary file 1 [file hcg-13-e002769-s001.pdf]

SUPPLEMENTAL MATERIAL

Supplemental methods

Discovery

The discovery meta-analysis contained 24 studies from CARDIoGRAM*plus*C4D, ENGAGE and SUMMIT consortia of European descent, except for PROMIS, which included individuals of South Asian descent for which full summary statistics were available (Supplementary Table 1). Cases were selected for inclusion following the standard criteria for CAD and myocardial infarction used in the CARDIoGRAM*plus*C4D consortium.[1] There was no sample overlap amongst the studies included from different consortia.

The discovery included both cross-sectional studies and longitudinal studies (analysed as cross-sectional studies). We accounted for the contemporaneous diagnoses of T2D and CAD in longitudinal studies by including CAD cases that had a diagnosis of T2D prior and up to 5 years after the CAD event.

To identify loci that were specific to CAD in the context of diabetes, and to identify loci that interacted with T2D to modify the risk of CAD, analyses were stratified by T2D status, which included 24,259 subjects with T2D (10,014 CAD cases) and 42,384 subjects without diabetes (17,694 CAD cases). Studies provided summary statistics for variants typed on the Cardio Metabochip array or provided GWAS data imputed to either HapMap2 or 1000 Genomes phase 1 reference panels.[2]

Replication

Replication was sought for loci that achieved a discovery *p* value$<1\times10^{-4}$ for association with CAD in at least one of the following analyses: all individuals combined regardless of T2D status; subjects with T2D only; subjects without diabetes; or the interaction analysis. Replication was conducted in 11,537 subjects with T2D (3,706 CAD cases) and 106,250 subjects without diabetes (12,988 CAD cases) from four studies of European descent (Supplementary Table 2). The samples used in the replication analyses were independent of those used in the discovery analysis.

Joint analysis and combination of evidence

We had access to full summary statistics for the discovery analysis and requested summary statistics for variants selected for replication from replication cohorts. Thus, we performed a joint analysis between the estimates for individual variants from the discovery analyses and summary statistics for a subset of variants selected for replication. Analyses were performed in each study to test the following comparisons: CAD in subjects with T2D and CAD in subjects without diabetes. Association was tested with CAD status in a regression model, adjusted for age, sex and study specific covariates such as principal components to account for population structure, where applicable. Age was defined as age of event for CAD cases and age at sampling for controls.

Genotype characteristics for the discovery cohort are given in Supplementary Table 3. We excluded variants: minor allele frequency (MAF)<1%; 2×N cases ×MAF<10; Hardy-Weinberg equilibrium test *p* value (p_{hwe}) $<5\times10^{-7}$ and MAF>5% or $p_{hwe}<1\times10^{-4}$ and MAF<5% for directly typed variants and imputation information score <0.4 (IMPUTE2)/imputation information score <0.3 (MaCH) for imputed variants or a call rate <95% for directly typed variants.

We used the additive model to generate association summary statistics and combined these statistics in a fixed-effect inverse variance-weighted meta-analysis using GWAMA v2.1.[3] We used a fixed effects model to estimate the allelic effects in individual strata, under the assumptions that any differences in allelic effect between strata were due to type 2 diabetes background. This method did not account for between study variation in allelic effects. To test for heterogeneous allelic effects by T2D status, we used the method outlined by Magi et al., 2010.[3] We double genomic control (GC) corrected association summary statistics both at the study level and in the overall discovery meta-analysis. Variants were excluded from the discovery meta-analysis if the effective sample size < 4000.

We estimated the interaction effects based on comparing the summary allelic effect on CAD for each variant between subjects with and without T2D. This approach allowed us to include more samples in the meta-analyses as studies that did not contain both subjects with and without T2D could be

included in the stratified analyses but would have been excluded from a meta-analysis of the interaction term. By adopting this approach, we were able to increase the sample size but were unable to account for between study variance in allelic effects.

Signal declaration criteria

We selected loci for replication based on a *p* value of association for the lead variant $\leq 1\times10^{-4}$ in the T2D only, non-diabetic only and interaction analyses. The genotype characteristics of the replication cohorts are given in Supplementary Table 4. We estimated the combined effect sizes for variants in a joint analysis based on a fixed-effect inverse variance–weighted meta-analysis using GWAMA v2.1.[3] Novel loci were declared at $p\leq 5\times10^{-8}$. For declaring interaction signals, we required that the directions of effect for individual strata were consistent across the discovery and replication analyses and achieved a $p_{interaction}<0.05/175$ (the number of variants selected for replication) in the replication analysis. Suggestive interaction signals were identified as those that showed directional consistency across the discovery and replication analyses, and when combined in joint analysis achieved combined $p_{interaction}$ < discovery $p_{interaction}$.

Power calculations

Stratified and overall analyses

Power calculations were conducted in R statistics using the gap package.[4] For the purpose of the power calculations effective population size was used (4/ (1/Ncases+1/Nctrls) and a disease prevalence of 5%. The calculation also considered allele frequency and effect size. We used an $\alpha\leq 5\times10^{-8}$ for novel loci.

Interaction analysis

Statistical interaction was calculated by testing the difference between two estimates of allelic effect on CAD. The allelic effects were estimated in subjects with diabetes and without diabetes separated and were compared using GWAMA v2.1 to calculate a $p_{interaction}$.[3] The power to detect an interaction

depends on how accurately the allelic effect can be estimated in each stratum. We assessed the power to detect an interaction effect of a CAD-risk variant with T2D in three allelic effect scenarios: a) an effect on CAD in subjects with T2D only (i.e. OR is 1 in subjects without diabetes, but varies between 1 and 1.2 in subjects with T2D); b) an effect on CAD in subjects with T2D and without diabetes in the same direction but of differing magnitude (i.e. OR is 1.10 in subjects without diabetes, but varies between 1 and 1.2 in subjects with T2D); and c) an effect on CAD in subjects with T2D and without diabetes but in opposite directions (i.e. OR is 0.90 in subjects without diabetes, but varies between 1 and 1.2 in subjects with T2D). For each scenario, we evaluated a range of risk allele frequencies: 10%, 20% and 50%. Power was calculated for $\alpha \leq 1 \times 10^{-4}$ in discovery (using discovery sample sizes) based on the threshold for replication.

Genetic correlation with related risk factors

We assessed the genetic correlation of CAD by diabetes subgroup with related risk factors using LDHub.[5] Genetic correlation was calculated by taking the slope of the regression of the product of trait 1 z scores on trait 2 z scores on the LD score for a SNP. Z scores were derived from the allelic effects and standard error for that trait. We restricted the analysis to 106 traits available in LDHub that are known risk factors for T2D and CAD.

Genetic risk score analysis

SNPs associated with Waist-Hip-ratio (adjusted for body mass index [BMI]), number of SNPs [N_{SNPs}]=53),[6] BMI (untransformed, N_{SNPs}=95 and z-transformed, N_{SNPs}=23),[7,8] systolic blood pressure (SBP) (N_{SNPs}=21),[9,10] LDL-C (N_{SNPs}=143), HDL-C (N_{SNPs}=143), triglycerides (N_{SNPs}=143),[11] T1D, T2D (N_{SNPs}=403),[12] 2-hr glucose (adjusted for BMI, N_{SNPs}=15),[13] fasting glucose (FG, adjusted for BMI, N_{SNPs}=21),[14] glycated haemoglobin (N_{SNPs}=15),[15] fasting insulin (natural log transformed and adjusted for BMI, N_{SNPs}=13),[14] fasting pro-insulin (adjusted for BMI and FG, N_{SNPs}=10),[16] HOMA-B(N_{SNPs}=15), HOMA-IR(N_{SNPs}=15)[17] and insulin resistance (N_{SNPs}=10)[18] at genome-wide significance ($p \leq 5 \times 10^{-8}$) were included in a genetic risk score (GRS) for each trait respectively. To account for the pleiotropic effects

amongst the lipid associated loci a multivariable model was employed from in R using the TwoSampleMR package.[19] For all other GRS the inverse variance weighted method was used to associate each of the GRS with CAD summary statistics.[20]

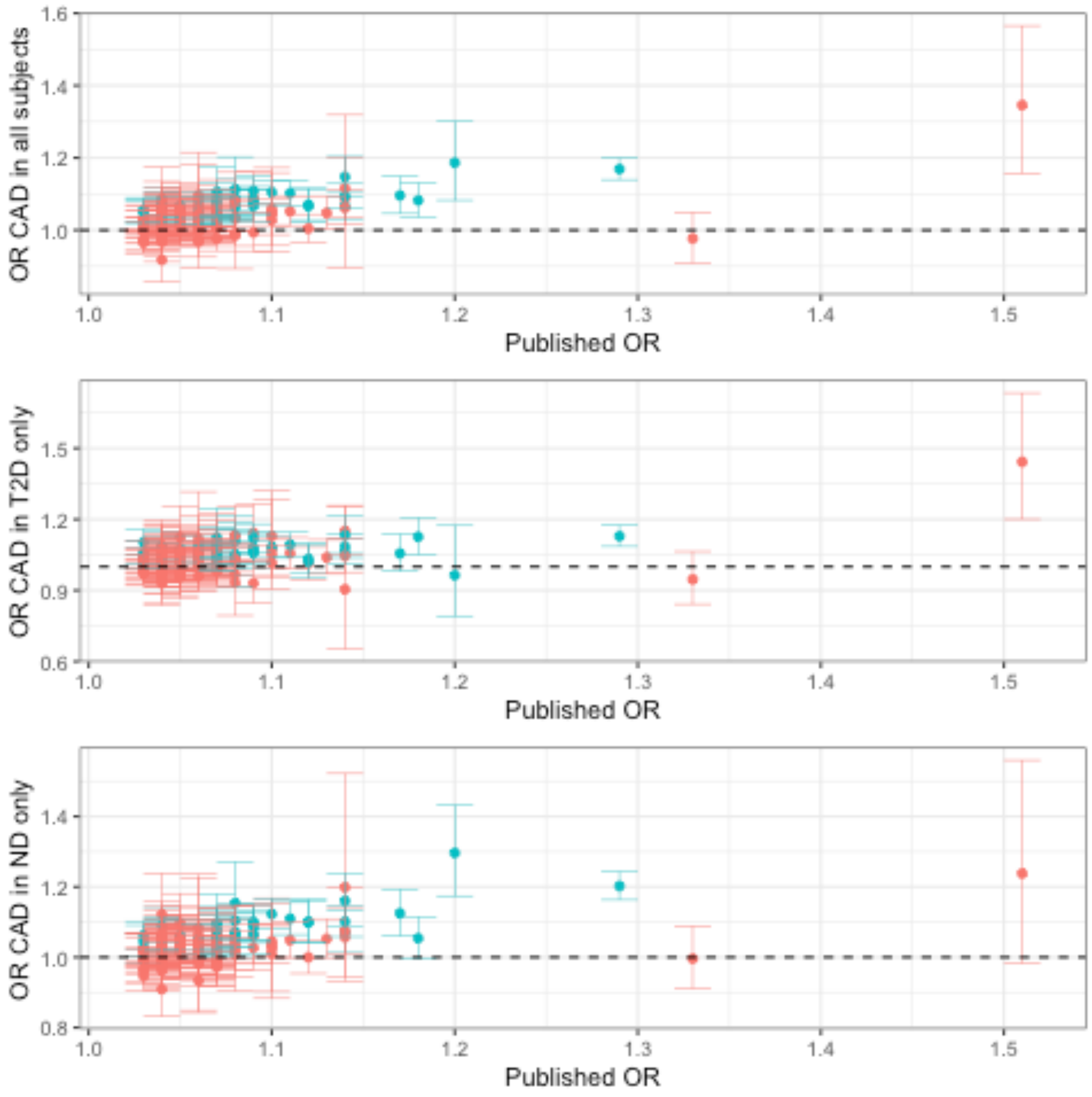

Supplementary Figure 1: Odds ratios (OR) for 160 known coronary artery disease (CAD) loci from this study compared to the published OR in the combined analysis of CAD, CAD in subjects with T2D and for CAD in subjects without diabetes. The blue colour indicates a p<5x10-3 in at least one of the analyses. For these SNPs the odds ratios >/= 1.00 for the published risk allele.

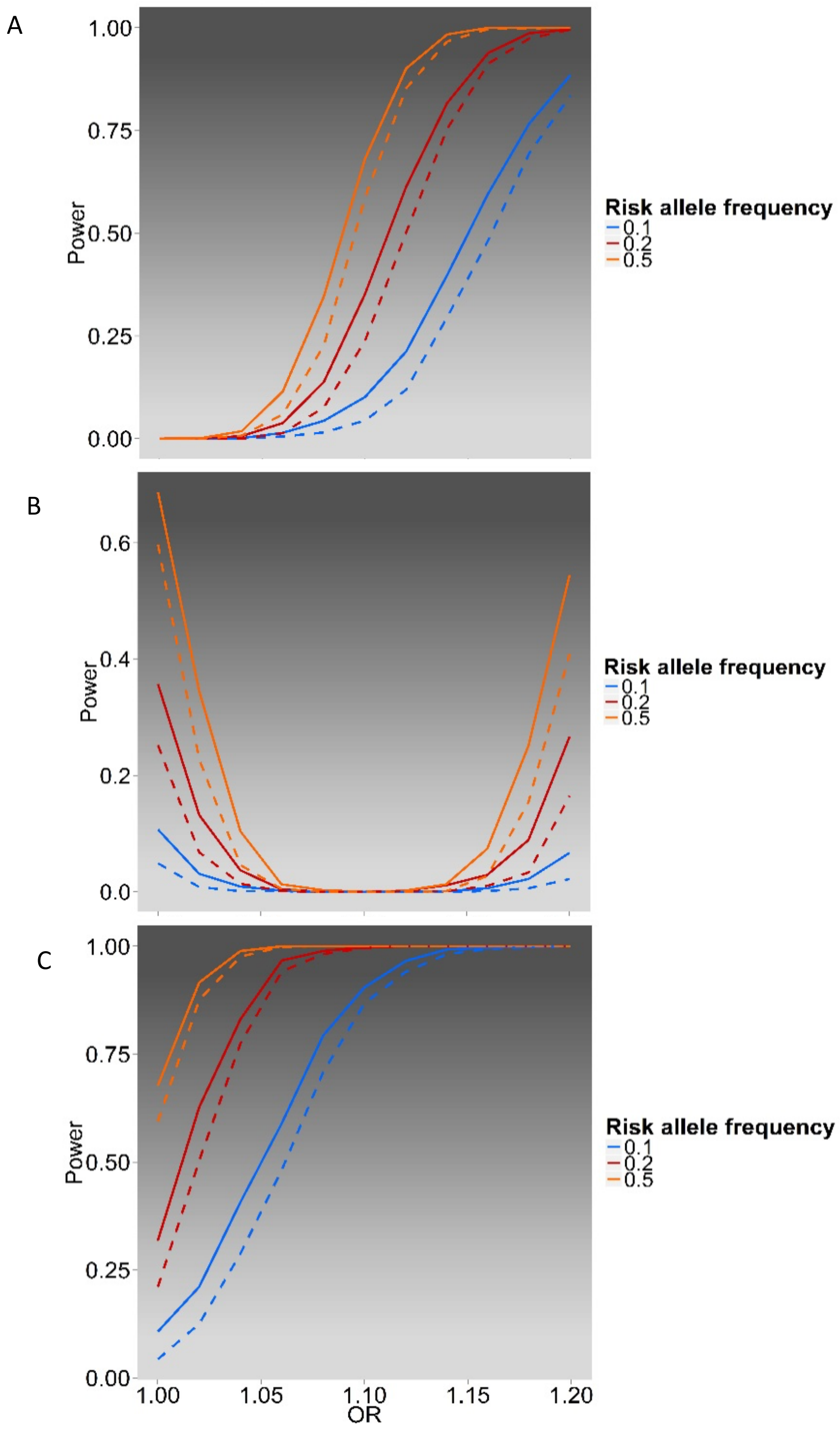

Supplementary Figure 2: Interactions can be broadly classified into three classes: A) effect on coronary artery disease (CAD) is specific to subjects with type 2 diabetes (T2D) (i.e. OR is 1.0 in diabetes free subjects, but varies between 1.0 and 1.2 in subjects with T2D); B) effect on CAD is heterogeneous, but is in the same direction irrespective of diabetes status (i.e. OR is 1.10 in diabetes free subjects, but varies between 1.0 and 1.2 in subjects with T2D); and C) effect on CAD is heterogeneous, and is in the opposite direction in subjects with T2D and diabetes free subjects (i.e. OR is 0.9 in diabetes free subjects, but varies between 1.0 and 1.2 in subjects with T2D). The continuous lines represent the power to detect an interaction $p<1\times10^{-4}$ in discovery and the dashed line a $p<0.05$ in the replication.

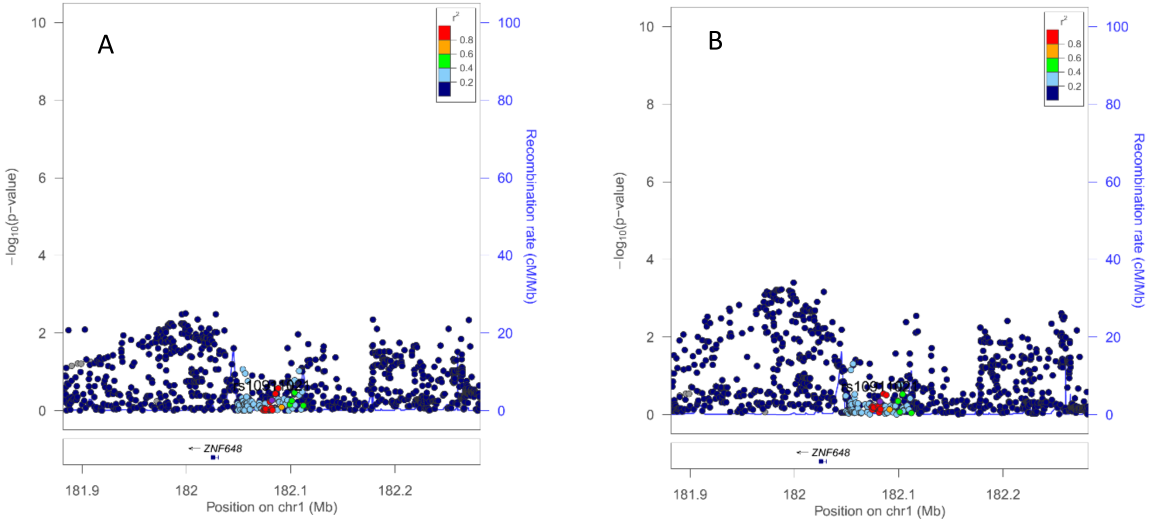

Supplementary Figure 3: Locuszoom plots of association statistics in the region of *ZNF648* for a published variant rs10911021 with coronary artery disease (A) and the interaction p value with type 2 diabetes (B). This variant has been previously reported to interact with T2D to modify the risk of coronary artery disease.

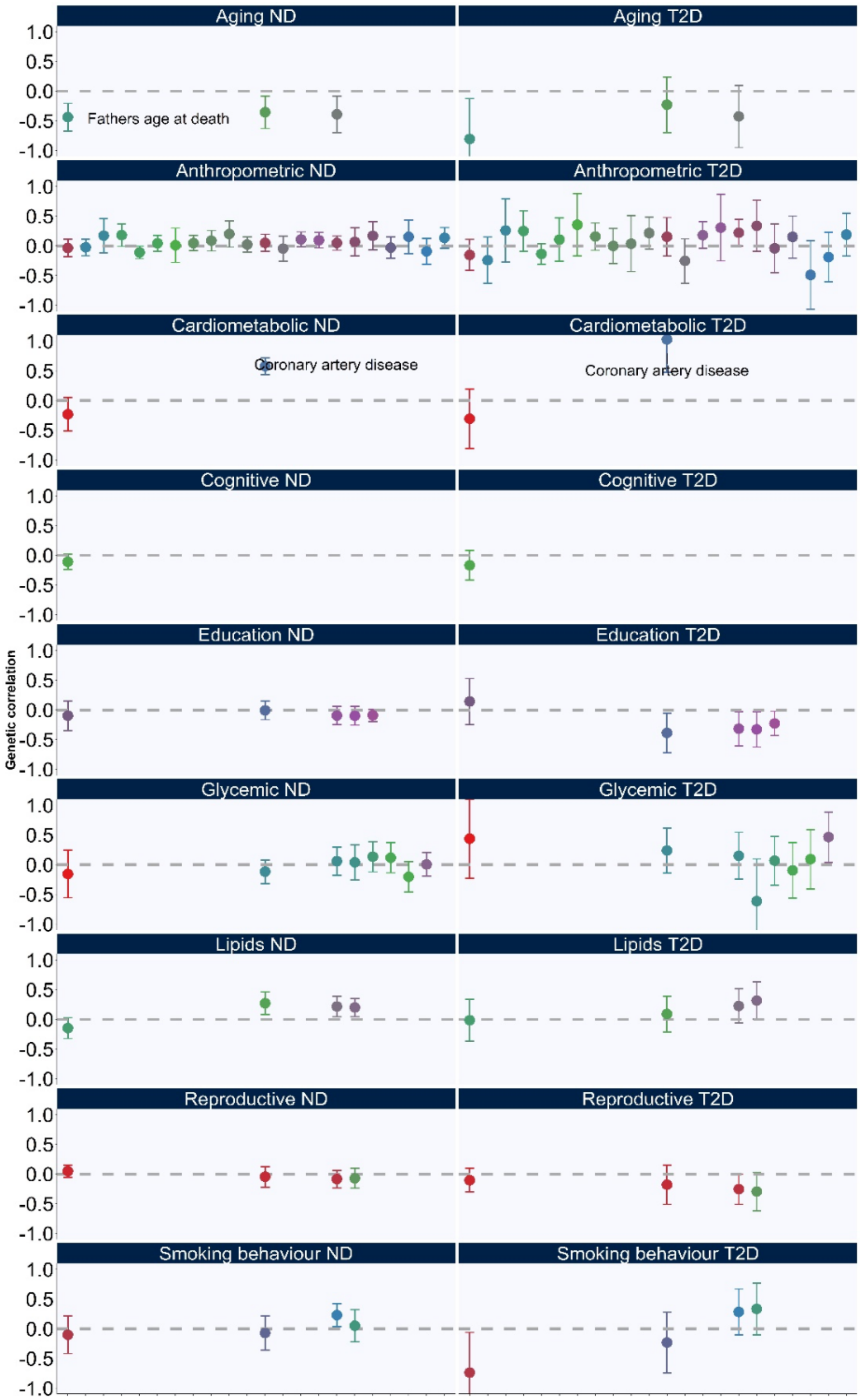

Supplementary Figure 4: Genetic correlation of coronary artery disease stratified by type 2 diabetes with known risk factors for CAD. Asterisks indicate a p value < 4.1×10^{-4} (0.05/121) for accuracy in the estimation of genetic correlation. Where the error bars do not cross zero-line p<0.05. Genetic correlations are calculated from all variants rather than those reaching genome-wide significance and give a broader picture of the overall genetic overlap. A different colour point is assigned to each trait.

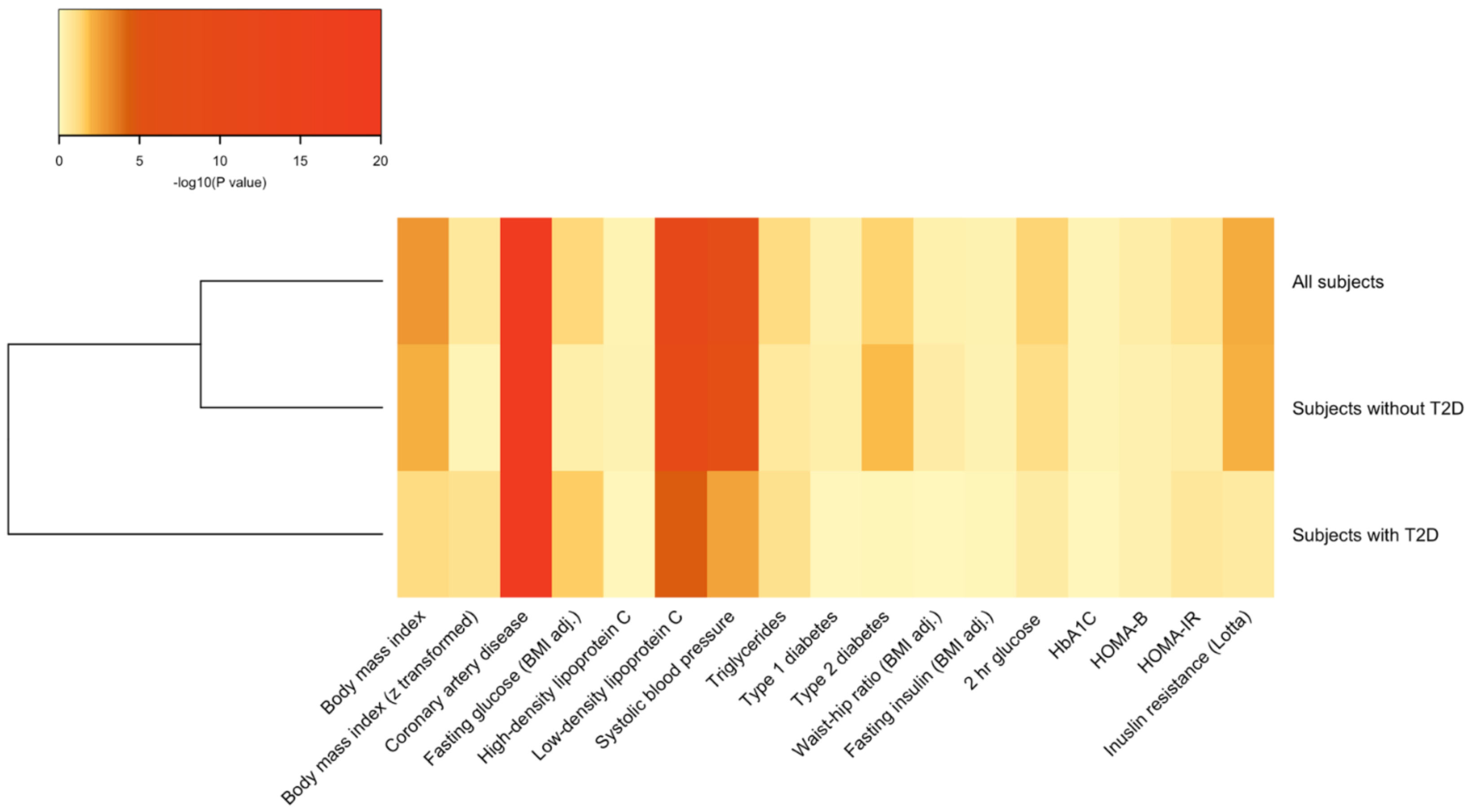

Supplementary Figure 5: The heat map of genetic risk scores for known coronary artery disease (CAD) risk factors does not show any significant differences between CAD in subjects with and without diabetes. Genetic risk scores (GRS) were for known CAD risk factors were constructed and associated with CAD in all individuals, CAD in subjects without T2D and with CAD in subjects with T2D. This was to determine if there was a different effect of risk factors on CAD by T2D background. Similar colours indicate a similar strength of association between the GRS for the known CAD risk factor and CAD in the different contexts examined.

Supplementary Table 1: The discovery meta-analysis included 24 studies and the sample characteristics of those studies are provided in the accompanying excel file.

Supplementary Table 2: Phenotypic characteristics of the 4 studies that were included in the replication meta-analyses

STUDY	HPFS	NHS	METSIM	DECODE
FULL NAME	The Health Professionals Follow-Up Study	The Nurses' Health Study	The Metabolic Syndrome In Men Study	deCODE Study
REFERENCE	PMID: 23982368	PMID: 23982368	PMID: 19223598	PMID: 27192541
ETHNICITY	Caucasian	Caucasian	European	European
REGION OF RECRUITMENT	The U.S.	The U.S.	Finland	Iceland
PHENOTYPE	CHD	CHD	CAD	CAD
PHENOTYPE DEFINITION	Nonfatal CHD was confirmed using the criteria of the World Health Organization, specifically, on the basis of symptoms and either electrocardiographic changes or elevated cardiac enzyme concentrations. Fatal CHD was defined as fatal myocardial infarction if this was confirmed by hospital records or autopsy, or if CHD was listed as the cause of death on the certificate and this was the underlying and only plausible cause, and evidence of previous CHD was available.	Nonfatal CHD was confirmed using the criteria of the World Health Organization, specifically, on the basis of symptoms and either electrocardiographic changes or elevated cardiac enzyme concentrations. Fatal CHD was defined as fatal myocardial infarction if this was confirmed by hospital records or autopsy, or if CHD was listed as the cause of death on the certificate and this was the underlying and only plausible cause, and evidence of previous CHD was available.	Hopsital admission codes and death records for: MI/Unstable Angina/Corrective procedures (PTCA & CABG)	Coronary artery disease (CAD) was defined as a) individuals in the MONICA registry who suffered myocardial infarction (MI) before the age of 75 in Iceland between 1981 and 2002 and satisfied the MONICA criteria1, b) subjects with CAD discharge diagnoses (ICD 9 codes 410.*, 411.*, 412.*, 414.* or ICD 10 codes I20.0, I21.*, I22.*. I23.*, I24.*, I25.*) from LUH, c) subjects diagnosed with significant angiographic CAD (see below) identified from a nationwide clinical registry of coronary

STUDY	HPFS	NHS	METSIM	DECODE
				**angiography and percutaneous coronary interventions at LUH between the years 1987 and 2012, d) subjects undergoing coronary artery bypass grafting (CABG) procedures at LUH between the years 2002 and 2011 and e) cause of death or contributing cause of death listed as MI or CAD (ICD 9 or 10 codes) on death registries between the years 1996 and 2009. Coronary angiograms in the nationwide registry were evaluated by an interventional cardiologist. Patients were considered to have significant angiographic CAD if one or more of the three major epicardial coronary vessels or the left main coronary artery was found to have at least 50% stenosis by visual estimation.**

STUDY	HPFS	NHS	METSIM	DECODE
CONTROL DEFINITION	CHD controls were selected randomly and matched in a 1:2 ratio on age, smoking, and month of blood return, among participants who were free of CVD and T2D when CHD was diagnosed in the case.	CHD controls were selected randomly and matched in a 1:2 ratio on age, smoking, and month of blood return, among participants who were free of CVD and T2D when CHD was diagnosed in the case.	Controls free of CAD	Population controls without known history of CAD.
MEAN AGE (SD) YEARS [CASES/CONTROLS] - AGE AT CATH			62.12 (6.40) / 57.07 (7.00)	66.9 (12.9) / 57.7 (17.6)
N CAD/MI [CASES/CONTROLS]	750/1403	644/1689	1173/8999	14156 / 89340
N DIABETIC CAD/MI [CASES/CONTROLS]	385/646	314/918	391/1579	2619 / 4712
N NON-DIABETIC CAD/MI CASES [CASES/CONTROLS]	365/756	330/771	782/7420	11537 / 84628
% EVER SMOKER CAD/MI [CASES/CONTROLS]			17.0%/18.2 %	54.3% / 38.8%
BMI DIABETIC CAD/MI [CASES/CONTROLS]			29.78 (4.64) / 29.90 (5.05)	29.3 (5.3) / 30.3 (6.1)
BMI NON-DIABETIC CAD/MI [CASES/CONTROLS]			27.01 (3.83) / 26.63 (3.71)	26.5 (4.4) / 26.5 (5.0)

Supplementary Table 3: The phenotypic characteristics of studies included in the discovery meta-analysis can be found in the accompanying excel file.

Supplementary Table 4: Genotypic characteristics of the four studies that were included in the replication analyses.

STUDY NAME	HPFS	NHS	METSIM	DECODE
GENOTYPING CENTRE	The Broad Center for Genotyping and Analysis	The Broad Center for Genotyping and Analysis	NHGRI	deCODE
GENOTYPING ARRAY	Affymetrix Genome-Wide Human 6.0 array	Affymetrix Genome-Wide Human 6.0 array	HumanOmniExpress-12v1 or HumanExome-12v1_A	Illumina HumanHap300, HumanCNV370, HumanHap610, HumanHap1M, HumanHap660, Omni-1, Omni 2.5 or Omni Express bead chips
CALLING ALGORITHM	the Birdseed calling algorithm	the Birdseed calling algorithm	GenomeStudio version 2011.1,Genotyping Module version 1.9.4, GenTrain version 1.0	GenomeStudio

STUDY NAME	HPFS	NHS	METSIM	DECODE
**PRE-IMPUTATION QC - EXCLUSION CRITERIA**	**Genotypic data first passed Broad's initial QC which included SNP fingerprints for sample tracking and early detection of sample misidentification, missing call rates of ≥ 5%, the use of a HapMap control to check genotype quality independent of study samples and tracking of reagent and instrumental performance**	**Genotypic data first passed Broad's initial QC which included SNP fingerprints for sample tracking and early detection of sample misidentification, missing call rates of ≥ 5%, the use of a HapMap control to check genotype quality independent of study samples and tracking of reagent and instrumental performance**		**Chip SNPs were excluded if they had (i) yield less than 95%, (ii) minor allele frequency (MAF) less than 1% in the population or (iii) significant deviation from Hardy-Weinberg equilibrium (P < 0.001), (iv) if they produced an excessive inheritance error rate (over 0.001), (v) if there was substantial difference in allele frequency between chip types (from just a single chip if that resolved all differences, but from all chips otherwise). All samples with a call rate below 97% were excluded from the**

STUDY NAME	HPFS	NHS	METSIM	DECODE
				analysis.Individuals not of Icelandic origin were excluded.
SAMPLE CALL RATE	>98%	>98%	99%	>97%
SNP CALL RATE	>99%	>99%	>95%	>95%
HWE			>0.00029	P > 0.001
IMPUTATION SOFTWARE	MACH	MACH		deCode's imputation pipeline
REFERENCE PANEL	NCBI build 37	NCBI build 37		2636 WGS Icelanders
ANALYSIS SOFTWARE	Plink	Plink	Plink	R
ANALYSIS MODEL	Logistic regression	Logistic regression	Logistic regression	Logistic regression
TOTAL SNPS INCLUDED IN ANALYSIS DIABETIC ONLY [GENOTYPED/IMPUTED]	456	456	290	646 / 0
TOTAL SNPS INCLUDED IN ANALYSIS NON-DIABETIC ONLY [GENOTYPED/IMPUTED]	548	548	330	646 / 0

Supplementary Table 5**:** Results from the T2D stratified analysis for SNPs in known coronary artery disease loci (N=160) in the combined, T2D only and non-diabetic analyses. This table shows the odds ratios from each of the analyses performed and reports the published odds ratio.

The table is available in the supplementary excel file

Supplementary Table 6: Pairwise genetic correlation between 106 traits and coronary artery disease (CAD) stratified by type 2 diabetes (T2D) status using data from LDScore hub. Genetic correlations indicate the overall genetic overlap between traits. Here we wanted to understand the genetic overlap of known CAD risk factors with CAD in the context of T2D.

Coronary Artery Disease	Category	Trait	PMID	rg	se	p
In subjects with diabetes	Aging	Fathers age at death	27015805	-0.81	0.35	0.02
In subjects without diabetes	Aging	Fathers age at death	27015805	-0.44	0.12	2.0×10^{-4}
In subjects with diabetes	Aging	Mothers age at death	27015805	-0.23	0.24	0.34
In subjects without diabetes	Aging	Mothers age at death	27015805	-0.36	0.14	0.01
In subjects with diabetes	Aging	Parents age at death	27015805	-0.43	0.27	0.11
In subjects without diabetes	Aging	Parents age at death	27015805	-0.39	0.16	0.01
In subjects with diabetes	Anthropometric	Birth weight	27680694	-0.15	0.13	0.25
In subjects without diabetes	Anthropometric	Birth weight	27680694	-0.04	0.08	0.64
In subjects with diabetes	Anthropometric	Body fat	26833246	0.15	0.17	0.36
In subjects without diabetes	Anthropometric	Body fat	26833246	0.05	0.07	0.50
In subjects with diabetes	Anthropometric	Body mass index	20935630	0.22	0.12	0.06
In subjects without diabetes	Anthropometric	Body mass index	20935630	0.05	0.06	0.44

Coronary Artery Disease	Category	Trait	PMID	rg	se	p
In subjects with diabetes	Anthropometric	Child birth length	25281659	0.34	0.22	0.13
In subjects without diabetes	Anthropometric	Child birth length	25281659	0.07	0.12	0.57
In subjects with diabetes	Anthropometric	Child birth weight	23202124	-0.04	0.21	0.84
In subjects without diabetes	Anthropometric	Child birth weight	23202124	0.17	0.12	0.16
In subjects with diabetes	Anthropometric	Childhood obesity	22484627	0.15	0.18	0.41
In subjects without diabetes	Anthropometric	Childhood obesity	22484627	-0.03	0.09	0.74
In subjects with diabetes	Anthropometric	Difference in height between adolescence and adulthood; age 14	23449627	-0.49	0.30	0.10
In subjects without diabetes	Anthropometric	Difference in height between adolescence and adulthood; age 14	23449627	0.15	0.15	0.30
In subjects with diabetes	Anthropometric	Difference in height between childhood and adulthood; age 8	23449627	-0.19	0.21	0.37
In subjects without diabetes	Anthropometric	Difference in height between childhood and adulthood; age 8	23449627	-0.09	0.11	0.40
In subjects with diabetes	Anthropometric	Extreme bmi	23563607	0.19	0.18	0.31
In subjects without diabetes	Anthropometric	Extreme bmi	23563607	0.13	0.09	0.13
In subjects with diabetes	Anthropometric	Extreme height	23563607	-0.24	0.20	0.22
In subjects without diabetes	Anthropometric	Extreme height	23563607	-0.03	0.07	0.73

Coronary Artery Disease	Category	Trait	PMID	rg	se	p
In subjects with diabetes	Anthropometric	Extreme waist-to-hip ratio	23563607	0.26	0.27	0.34
In subjects without diabetes	Anthropometric	Extreme waist-to-hip ratio	23563607	0.17	0.15	0.24
In subjects with diabetes	Anthropometric	Height 2010	20881960	-0.14	0.09	0.13
In subjects without diabetes	Anthropometric	Height 2010	20881960	-0.11	0.05	0.04
In subjects with diabetes	Anthropometric	Height; Females at age 10 and males at age 12	23449627	0.25	0.17	0.15
In subjects without diabetes	Anthropometric	Height; Females at age 10 and males at age 12	23449627	0.18	0.10	0.06
In subjects with diabetes	Anthropometric	Hip circumference	25673412	0.11	0.19	0.57
In subjects without diabetes	Anthropometric	Hip circumference	25673412	0.04	0.07	0.52
In subjects with diabetes	Anthropometric	Infant head circumference	22504419	0.35	0.27	0.18
In subjects without diabetes	Anthropometric	Infant head circumference	22504419	0.01	0.15	0.95
In subjects with diabetes	Anthropometric	Obesity class 1	23563607	0.16	0.12	0.18
In subjects without diabetes	Anthropometric	Obesity class 1	23563607	0.05	0.07	0.48
In subjects with diabetes	Anthropometric	Obesity class 2	23563607	0.00	0.15	0.99
In subjects without diabetes	Anthropometric	Obesity class 2	23563607	0.09	0.09	0.32

Coronary Artery Disease	Category	Trait	PMID	rg	se	p
In subjects with diabetes	Anthropometric	Obesity class 3	23563607	0.04	0.24	0.88
In subjects without diabetes	Anthropometric	Obesity class 3	23563607	0.20	0.11	0.08
In subjects with diabetes	Anthropometric	Overweight	23563607	0.21	0.14	0.12
In subjects without diabetes	Anthropometric	Overweight	23563607	0.02	0.07	0.73
In subjects with diabetes	Anthropometric	Sitting height ratio	25865494	-0.25	0.19	0.18
In subjects without diabetes	Anthropometric	Sitting height ratio	25865494	-0.05	0.11	0.67
In subjects with diabetes	Anthropometric	Waist circumference	25673412	0.31	0.29	0.28
In subjects without diabetes	Anthropometric	Waist circumference	25673412	0.09	0.07	0.15
In subjects with diabetes	Anthropometric	Waist-to-hip ratio	25673412	0.18	0.11	0.12
In subjects without diabetes	Anthropometric	Waist-to-hip ratio	25673412	0.11	0.06	0.09
In subjects with diabetes	Cardiometabolic	Adiponectin	22479202	-0.31	0.26	0.23
In subjects without diabetes	Cardiometabolic	Adiponectin	22479202	-0.23	0.14	0.11
In subjects with diabetes	Cardiometabolic	Coronary artery disease	26343387	1.03	0.28	3.0×10^{-4}
In subjects without diabetes	Cardiometabolic	Coronary artery disease	26343387	0.58	0.07	1.1×10^{-16}

Coronary Artery Disease	Category	Trait	PMID	rg	se	p
In subjects with diabetes	Cognitive	Intelligence	28530673	-0.17	0.13	0.19
In subjects without diabetes	Cognitive	Intelligence	28530673	-0.11	0.06	0.09
In subjects with diabetes	Education	Childhood IQ	23358156	0.14	0.20	0.47
In subjects without diabetes	Education	Childhood IQ	23358156	-0.10	0.13	0.45
In subjects with diabetes	Education	College completion	23722424	-0.39	0.17	0.02
In subjects without diabetes	Education	College completion	23722424	-0.01	0.08	0.92
In subjects with diabetes	Education	Years of schooling (proxy cognitive performance)	25201988	-0.32	0.15	0.03
In subjects without diabetes	Education	Years of schooling (proxy cognitive performance)	25201988	-0.09	0.08	0.22
In subjects with diabetes	Education	Years of schooling 2013	23722424	-0.33	0.15	0.03
In subjects without diabetes	Education	Years of schooling 2013	23722424	-0.10	0.08	0.21
In subjects with diabetes	Education	Years of schooling 2016	27225129	-0.23	0.10	0.03
In subjects without diabetes	Education	Years of schooling 2016	27225129	-0.09	0.05	0.09
In subjects with diabetes	Glycemic	2hr glucose adjusted for BMI	20081857	0.44	0.34	0.20
In subjects without diabetes	Glycemic	2hr glucose adjusted for BMI	20081857	-0.16	0.20	0.44

Coronary Artery Disease	Category	Trait	PMID	rg	se	p
In subjects with diabetes	Glycemic	Fasting glucose main effect	22581228	0.24	0.19	0.22
In subjects without diabetes	Glycemic	Fasting glucose main effect	22581228	-0.12	0.10	0.25
In subjects with diabetes	Glycemic	Fasting insulin main effect	22581228	0.15	0.20	0.46
In subjects without diabetes	Glycemic	Fasting insulin main effect	22581228	0.06	0.12	0.62
In subjects with diabetes	Glycemic	Fasting proinsulin	20081858	-0.62	0.36	0.09
In subjects without diabetes	Glycemic	Fasting proinsulin	20081858	0.04	0.15	0.79
In subjects with diabetes	Glycemic	HbA1C	20858683	0.07	0.21	0.75
In subjects without diabetes	Glycemic	HbA1C	20858683	0.14	0.13	0.30
In subjects with diabetes	Glycemic	HOMA-B	20081858	-0.09	0.24	0.69
In subjects without diabetes	Glycemic	HOMA-B	20081858	0.12	0.13	0.36
In subjects with diabetes	Glycemic	HOMA-IR	20081858	0.09	0.26	0.72
In subjects without diabetes	Glycemic	HOMA-IR	20081858	-0.20	0.13	0.12
In subjects with diabetes	Glycemic	Type 2 Diabetes	22885922	0.46	0.22	0.03
In subjects without diabetes	Glycemic	Type 2 Diabetes	22885922	0.01	0.10	0.96

Coronary Artery Disease	Category	Trait	PMID	rg	se	p
In subjects with diabetes	Lipids	HDL cholesterol	20686565	-0.01	0.18	0.94
In subjects without diabetes	Lipids	HDL cholesterol	20686565	-0.15	0.09	0.10
In subjects with diabetes	Lipids	LDL cholesterol	20686565	0.09	0.15	0.56
In subjects without diabetes	Lipids	LDL cholesterol	20686565	0.27	0.10	0.01
In subjects with diabetes	Lipids	Total Cholesterol	20686565	0.23	0.15	0.12
In subjects without diabetes	Lipids	Total Cholesterol	20686565	0.22	0.09	0.01
In subjects with diabetes	Lipids	Triglycerides	20686565	0.32	0.16	0.05
In subjects without diabetes	Lipids	Triglycerides	20686565	0.20	0.08	0.01
In subjects with diabetes	Reproductive	Age at Menarche	25231870	-0.10	0.10	0.31
In subjects without diabetes	Reproductive	Age at Menarche	25231870	0.05	0.05	0.35
In subjects with diabetes	Reproductive	Age at Menopause	26414677	-0.18	0.17	0.29
In subjects without diabetes	Reproductive	Age at Menopause	26414677	-0.05	0.09	0.61
In subjects with diabetes	Reproductive	Age of first birth	27798627	-0.25	0.13	0.05
In subjects without diabetes	Reproductive	Age of first birth	27798627	-0.08	0.07	0.27

Coronary Artery Disease	Category	Trait	PMID	rg	se	p
In subjects with diabetes	Reproductive	Number of children ever born	27798627	-0.29	0.16	0.07
In subjects without diabetes	Reproductive	Number of children ever born	27798627	-0.07	0.09	0.40
In subjects with diabetes	Smoking behaviour	Age of smoking initiation	20418890	-0.74	0.35	0.03
In subjects without diabetes	Smoking behaviour	Age of smoking initiation	20418890	-0.10	0.16	0.53
In subjects with diabetes	Smoking behaviour	Cigarettes smoked per day	20418890	-0.23	0.26	0.37
In subjects without diabetes	Smoking behaviour	Cigarettes smoked per day	20418890	-0.07	0.15	0.63
In subjects with diabetes	Smoking behaviour	Ever vs never smoked	20418890	0.28	0.20	0.15
In subjects without diabetes	Smoking behaviour	Ever vs never smoked	20418890	0.23	0.10	0.02
In subjects with diabetes	Smoking behaviour	Former vs Current smoker	20418890	0.34	0.22	0.13
In subjects without diabetes	Smoking behaviour	Former vs Current smoker	20418890	0.05	0.14	0.71

Supplementary Table 7: Coronary artery disease (CAD) has several risk factors that are in part genetically determined. We constructed genetic risk scores for known CAD risk factors and associated them with CAD by type 2 diabetes status.

CAD score	Risk Factor	OR	L95	U95	P
All subjects	Body mass index	1.06	1.02	1.09	1.6×10^{-3}
Subjects with T2D	Body mass index	1.05	1.00	1.10	0.07
Subjects without T2D	Body mass index	1.06	1.02	1.11	7.1×10^{-3}
All subjects	Body mass index (z transformed)	1.08	0.95	1.22	0.23
Subjects with T2D	Body mass index (z transformed)	1.16	0.96	1.41	0.12
Subjects without T2D	Body mass index (z transformed)	1.03	0.88	1.20	0.75
All subjects	Coronary artery disease	2.12	2.01	2.24	6.4×10^{-171}
Subjects with T2D	Coronary artery disease	2.05	1.88	2.23	1.1×10^{-59}
Subjects without T2D	Coronary artery disease	2.18	2.04	2.33	8.5×10^{-117}
All subjects	Fasting glucose (BMI adj.)	1.56	1.00	2.42	0.05
Subjects with T2D	Fasting glucose (BMI adj.)	2.13	1.08	4.21	0.03
Subjects without T2D	Fasting glucose (BMI adj.)	1.23	0.70	2.18	0.47
All subjects	High-density lipoprotein C	0.97	0.86	1.10	0.66
Subjects with T2D	High-density lipoprotein C	0.99	0.84	1.17	0.89
Subjects without T2D	High-density lipoprotein C	0.96	0.83	1.12	0.62
All subjects	Low-density lipoprotein C	1.37	1.25	1.50	1.0×10^{-11}
Subjects with T2D	Low-density lipoprotein C	1.32	1.16	1.50	3.3×10^{-5}
Subjects without T2D	Low-density lipoprotein C	1.43	1.28	1.60	6.6×10^{-10}
All subjects	Systolic blood pressure	1.66	1.39	1.98	1.7×10^{-8}
Subjects with T2D	Systolic blood pressure	1.58	1.16	2.16	3.7×10^{-3}
Subjects without T2D	Systolic blood pressure	1.70	1.37	2.10	9.9×10^{-7}
All subjects	Triglycerides	1.14	0.99	1.30	0.07
Subjects with T2D	Triglycerides	1.16	0.96	1.41	0.12

CAD score	Risk Factor	OR	L95	U95	P
Subjects without T2D	Triglycerides	1.11	0.93	1.33	0.26
All subjects	Type 1 diabetes	1.00	0.99	1.02	0.53
Subjects with T2D	Type 1 diabetes	1.00	0.98	1.02	0.95
Subjects without T2D	Type 1 diabetes	1.01	0.99	1.02	0.46
All subjects	Type 2 diabetes	1.04	1.00	1.08	0.04
Subjects with T2D	Type 2 diabetes	0.99	0.94	1.05	0.85
Subjects without T2D	Type 2 diabetes	1.06	1.01	1.11	0.01
All subjects	Waist-hip ratio (BMI adj.)	0.80	0.41	1.57	0.51
Subjects with T2D	Waist-hip ratio (BMI adj.)	1.01	0.36	2.82	0.99
Subjects without T2D	Waist-hip ratio (BMI adj.)	0.66	0.27	1.60	0.36
All subjects	Fasting insulin (BMI adj.)	1.17	0.64	2.14	0.62
Subjects with T2D	Fasting insulin (BMI adj.)	1.12	0.44	2.84	0.82
Subjects without T2D	Fasting insulin (BMI adj.)	1.20	0.55	2.63	0.65
All subjects	2 hr glucose	0.89	0.80	1.00	0.04
Subjects with T2D	2 hr glucose	0.91	0.76	1.09	0.31
Subjects without T2D	2 hr glucose	0.89	0.77	1.02	0.08
All subjects	HbA1C	1.09	0.69	1.70	0.72
Subjects with T2D	HbA1C	1.02	0.50	2.09	0.95
Subjects without T2D	HbA1C	1.12	0.64	1.98	0.69
All subjects	HOMA-B	1.18	0.82	1.70	0.38
Subjects with T2D	HOMA-B	1.21	0.68	2.15	0.52
Subjects without T2D	HOMA-B	1.15	0.72	1.84	0.55
All subjects	HOMA-IR	0.67	0.39	1.17	0.16
Subjects with T2D	HOMA-IR	0.55	0.22	1.40	0.21
Subjects without T2D	HOMA-IR	0.75	0.38	1.47	0.40
All subjects	Insulin resistance (Lotta)	1.01	1.00	1.01	6.3×10^{-3}
Subjects with T2D	Insulin resistance (Lotta)	1.00	1.00	1.01	0.28

CAD score	Risk Factor	OR	L95	U95	P
Subjects without T2D	Insulin resistance (Lotta)	1.01	1.00	1.01	0.01

Supplementary Table 8: CARDIoGRAM*plus*C4D Steering committee members

John Danesh

Department of Public Health and Primary Care, University of Cambridge, UK

Panos Deloukas

William Harvey research Institute, Barts and the London School of medicine & Dentistry, Queen Mary University of London, UK; Wellcome Trust Sanger Institute, Cambridge, UK

Jeanette Erdmann

Institut für Integrative und Experimentelle Genomik, Universität zu Lübeck, Lübeck, Germany

Dongfeng Gu

Department of Population Genetics and Prevention, Fu Wai Hospital & Cardiovascular Institute, Chinese Academy of Medical Sciences & Peking Union Medical College, Beijing, China

Anders Hamsten

Atherosclerosis Research Unit, Department of Medicine Solna, Karolinska Institutet, Stockholm, Sweden Center for Molecular Medicine and Department of Cardiology, Karolinska University Hospital, Stockholm, Sweden

Sekar Kathiresan

Cardiovascular Research Center and Center for Human Genetic Research, Massachusetts General Hospital, Boston, Massachusetts, USA; Broad Institute, Cambridge, Massachusetts, USA; Department of Medicine, Harvard Medical School, Boston, Massachusetts, USA

Jaspal S Kooner

National Heart and Lung Institute (NHLI), Imperial College London, Hammersmith Hospital, London, UK.

Robert Roberts

University of Ottawa Heart Institute, Cardiovascular Research Methods Centre and Ruddy Canadian Cardiovascular Genetics Centre, Ontario, Canada

Nilesh J Samani (Chair)

Department of Cardiovascular Sciences, University of Leicester, Glenfield Hospital, Leicester, UK; National Institute for Health Research (NIHR) Leicester Cardiovascular Biomedical Research Unit, Glenfield Hospital, Leicester, UK.

Heribert Schunkert

Deutsches Herzzentrum München, Technische Universität München, Munich, Germany

Unnur Thorsteinsdottir

deCODE Genetics, Sturlugata 8, Reykjavik, Iceland; University of Iceland, Faculty of Medicine, Reykjavik, Iceland

Hugh Watkins

Wellcome Centre for Human Genetics, University of Oxford, Oxford, UK; Cardiovascular Medicine, Radcliffe Department of Medicine, University of Oxford, Oxford, UK

Themistocles L Assimes

Department of Medicine, Stanford University School of Medicine, Stanford, California, USA

Email: tassimes@stanford.edu

Stefan Blankenberg

University Heart Center Hamburg, Clinic for general and interventional Cardiology, Hamburg, Germany

Bernhard O Boehm

Division of Endocrinology and Diabetes, Department of Internal Medicine, Ulm University Medical Centre, Ulm, Germany

John C Chambers

Epidemiology and Biostatistics, Imperial College London, London, UK

Robert Clarke

Clinical Trial Service Unit and Epidemiological Studies Unit, University of Oxford, Oxford, UK

Rory Collins

Clinical Trial Service Unit and Epidemiological Studies Unit, University of Oxford, Oxford, UK

George Dedoussis

Department of Dietetics-Nutrition, Harokopio University, Athens, Greece

Paul W Franks

Department of Clinical Sciences, Genetic & Molecular Epidemiology Unit, Lund University Diabetes Center, Skåne University Hosptial, Malmö, Sweden Department of Public Health & Clinical Medicine, Genetic Epidemiology & Clinical Research Group, Section for Medicine, Umeå University, Umeå, Sweden; Department of Nutrition, Harvard School of Public Health, Boston, MA USA

G Kees Hovingh

Department of Vascular Medicine, Academic Medical Center, Amsterdam, The Netherlands

Erik Ingelsson

Department of Medical Epidemiology and Biostatistics, Karolinska Institutet, Stockholm, Sweden

Bong-Jo Kim

Division of Structural and Functional Genomics Center for Genome Science, Korea National Institute of Health, Korea

Terho Lehtimäki

Department of Clinical Chemistry, Fimlab Laboratories, Tampere University Hospital, Tampere, Finland; Department of Clinical Chemistry, University of Tampere School of Medicine, Tampere, Finland

Winfried März

Synlab Academy, Mannheim, Germany; Medical Clinic V (Nephrology • Hypertensiology • Endocrinology • Diabetology • Rheumatology), Medical Faculty of Mannheim, University of Heidelberg, Mannheim, Germany

Ruth McPherson

Department of Medicine, Division of Cardiology, University of Ottawa Heart Institute, Ottawa, Canada

Andres Metspalu

Estonian Genome Center, University of Tartu, Tartu, Estonia; Institute of Molecular and Cell Biology, University of Tartu, Tartu, Estonia

Markku S Nieminen

Division of Cardiology, Department of Medicine, Helsinki University Central Hospital (HUCH), Helsinki, Finland

Christopher O'Donnell

Framingham Heart Study, National Heart, Lung and Blood Institute, Framingham, MA 01702, USA

Colin N A Palmer

Medical Research Institute, University of Dundee, Ninewells Hospital and Medical School, Dundee, UK

Markus Perola

Department of Chronic Disease Prevention, National Institute for Health and Welfare, Helsinki, Finland

Muredach P Reilly

Cardiovascular Institute, Perelman School of Medicine at the University of Pennsylvania, Philadelphia, USA

Samuli Ripatti

Department of Chronic Disease Prevention, National Institute for Health and Welfare, Helsinki FIN-00271, Finland; Wellcome Trust Sanger Institute, Hinxton, UK

Danish Saleheen

Center for Non-Communicable Diseases, Karachi, Pakistan; Department of Public Health and Primary Care, University of Cambridge, Cambridge, UK; Department of Medicine, University of Pennsylvania, Philadelphia, Pennsylvania, USA

Manjinder S Sandhu

MRC Epidemiology Unit, Institute of Metabolic Science, Addenbrooke's Hospital, Cambridge, UK; Wellcome Trust Sanger Institute, Hinxton, UK

Stefan Schreiber

Institut für Klinische Molekularbiologie, Christian-Albrechts Universität, Kiel, Germany

Agneta Siegbahn

Uppsala Clinical Research Center, Uppsala University, Uppsala, Sweden

Cristen J Willer

Department of Internal Medicine , Division of Cardiovascular Medicine, and Department of Human Genetics, University of Michigan, Ann Arbor, Michigan, USA

Pierre A Zalloua

Lebanese American University, Chouran, Beirut, Lebanon

Supplementary Table 9: Members of the SUMMIT consortium

Partner	Name	Position
1	Michael Mark	Coordinator, WP6 leader
Boehringer-Ingelheim	Markus Albertini	Project manager
Ingelheim, Germany	Carine Boustany	Chronic Kidney Disease, Head of Lab
	Alexander Ehlgen	Transmed
	Martin Gerl	Biomarker & Bioanlysis, Groupleader
	Jochen Huber	In vivo Scientist CMDR, Head of Lab
	Corinna Schölch	Biomarker & Bioanlysis, Head of Lab
	Heike Zimdahl-Gelling	Pharmacogenomics, Head of Lab
2	Leif Groop	Prof. Endocrinology; Coordinator Managing entity IMI-JU; PI; WP1 and WP6 leader
Lund University	Elisabet Agardh	Prof. Ophthalmology
Clinical Research Centre	Emma Ahlqvist	Postdoc
Malmö, Sweden	Tord Ajanki	Communication strategist
	Nibal Al Maghrabi	Research nurse
	Peter Almgren	Biostatistician
	Jan Apelqvist	Diabetologist
	Eva Bengtsson	Assis. Prof. Cardiovascular research
	Lisa Berglund	Postdoc
	Harry Björckbacka	Assis. Prof. Cardiovascular research
	Ulrika Blom-Nilsson	LUDC administrator
	Mattias Borell	Website, server management
	Agneta Burström	Research nurse
	Corrado Cilio	Assoc. Prof. Cellular autoimmunity
	Magnus Cinthio	Assist. Prof. Electrical Measurements, Lund Technical University
	Karl Dreja	Nephrologist
	Pontus Dunér	Postdoc Exp. Cardiovasc. Research
	Daniel Engelbertsen	PhD student Exp. Cardiovasc. Research
	Joao Fadista	Postdoc
	Maria Gomez	Assoc. Prof. Cardiovascular disease, WP4 co-leader
	Isabel Goncalves	Assis. Prof. Cardiovascular research
	Bo Hedblad	Prof. Cardiovascular epidemiology
	Anna Hultgårdh	Prof. Vessel Wall Biology
	Martin E. Johansson	Pathologist
	Cecilia Kennbäck	Laboratory Engineer
	Jasmina Kravic	Database manager
	Claes Ladenvall	Genetic statistician
	Åke Lernmark	Prof. Type 1 diabetes and celiac disease
	Eero Lindholm	Physician, Researcher Diabetic Complications

	**Charlotte Ling**	**Assist. Prof. Epigenetics**
	**Holger Luthman**	**Prof. Medical genetics**
	**Olle Melander**	**Assoc. Prof. Hypertension and cardiovascular disease**
	**Malin Neptin**	**Biomedical analyst**
	**Jan Nilsson**	**Prof. Experimental Cardiovascular research, WP3 leader**
	**Peter Nilsson**	**Prof. Internal medicine**
	**Tobias Nilsson**	**PhD student Electrical Measurements, Lund Technical University**
	**Gunilla Nordin Fredriksson**	**Prof. Cardiovascular research**
	**Marju Orho-Melander**	**Prof. Genetic epidemiology**
	**Emilia Ottoson-Laakso**	**PhD student**
	**Annie Persson**	**Research nurse**
	**Margaretha Persson**	**Laboratory Engineer**
	**Mats-Åke Persson**	**Database manager**
	**Jacqueline Postma**	**Project manager**
	**Elisabeth Pranter**	**Research nurse**
	**Sara Rattik**	**PhD student Exp. Cardiovasc. Research**
	**Gunnar Sterner**	**Chief physician Internal Medicine Research Unit**
	**Lilian Tindberg**	**Research nurse**
	**Maria Wigren**	**Postdoc Exp. Cardiovasc. Research**
	**Anna Zetterqvist**	**PhD student**
	**Mikael Åkerlund**	**Postdoc**
	**Gerd Östling**	**Laboratory Engineer**
**3**	**Timo Kanninen**	**Technical director; PI**
**Biocomputing Platforms**	**Anni Ahonen-Bishopp**	**Software development manager**
**(BC Platforms)**	**Anita Eliasson**	**Financial and administrative director**
**Espoo, Finland**	**Timo Herrala**	**System (server) specialist**
	**Päivi Tikka-Kleemola**	**Service manager**
**4**	**Anders Hamsten**	**Prof. Cardiovascular disease; Atherosclerosis Research Unit; PI**
**Karolinska Institute**	**Christer Betsholtz**	**Prof. Vascular biology**
**Stockholm, Sweden**	**Ami Björkholm**	**Administrator**
	**Ulf de Faire**	**Professor emeritus Cardiovascular epidemiology**
	**Fariba Foroogh**	**Research engineer**
	**Guillem Genové**	**Scientist**
	**Karl Gertow**	**Research Assist. Prof. Cardiovascular genetics**
	**Bruna Gigante**	**Assoc. Professor Cardiovascular epidemiology**
	**Bing He**	**Postdoc**
	**Karin Leander**	**Assoc. Professor Cardiovascular epidemiology**
	**Olga McLeod**	**Postdoc**

	Maria Nastase-Mannila	Postdoc
	Jaako Patrakka	Postdoc
	Angela Silveira	Assoc. Prof. Cardiovascular genetics
	Rona Strawbridge	Postdoc
	Karl Tryggvason	Prof. Medical Chemistry
	Max Vikström	Statistician
	John Öhrvik	Professor
	Anne-May Österholm	Postdoc
5	Barbara Thorand	Nutritional scientist, epidemiologist
Helmholtz Centre	Christian Gieger	Statistician
Munich, Germany	Harald Grallert	Biologist
	Tonia Ludwig	Statistician
	Barbara Nitz	Scientist
	Andrea Schneider	Data manager
	Rui Wang-Sattler	Scientist
	Astrid Zierer	Statistician
6	Giuseppe Remuzzi	Institute director; PI
Mario Negri Institute for	Ariela Benigni	Head of department Molecular Medicine
Pharmacological Research	Roberta Donadelli	Scientist
	Maria Domenica Lesti	Researcher
Bergamo, Italy	Marina Noris	Head Laboratory Immunology and genetics of transplantation and rare diseases
	Norberto Perico	Senior scientist
	Annalisa Perna	Biostatistician
	Rossella Piras	Postdoc
	Piero Ruggenenti	Head of department Renal medicine, Assist. Prof. Nephrology and dialysis
	Erica Rurali	Postdoc
7	David Dunger (att: Jane Horsford)	Prof. Paediatrics; PI
University of Cambridge	Ludo Chassin	Senior Data Manager
UK	Neil Dalton, London	Clinical biochemistry
	John Deanfield, London	Paediatric cardiology
	Jane Horsford	PA to Prof. Dunger
	Clare Rice	Operations manager/financial contact
	James Rudd	Cardiovascular imaging
	Neil Walker	Head Data services
	Karen Whitehead	Technician

	Max Wong	Postdoc
8	Helen Colhoun	Prof. Public health and epidemiology; PI; Vice coordinator Managing entity; WP2 leader
	Fiona Adams	
University of Dundee	Tahira Akbar	PA to Helen Colhoun
Scotland	Jill Belch	Prof. Vasucular disease
	Harshal Deshmukh	PhD student
	Fiona Dove	
	Angela Ellingford	NHS Tayside Diabetic Retinopathy Screening Programme manager
	Bassam Farran	Statistician
	Mike Ferguson	Dean of research Biological chemistry and drug discovery
	Gary Henderson	
	Graeme Houston	Consultant radiologist/senior lecturer
	Faisel Khan	Reader, Vascular & Inflammatory Diseases Research Unit
	Graham Leese	Consultant diabetologist/reader
	Yiyuan Liu	PhD student
	Shona Livingstone	Senior statistician
	Helen Looker	Epidemiologist
	Margaret McCann	Project assistant
	Stuart McGurnaghan	Lead data programmer
	David Newton	
	Colin Palmer	Prof. Pharmacogenomics
	Ewan Pearson	Consultant diabetologist/senior lecturer
	Gillian Reekie	Research Nurse
	Natalie Smith	Research Nurse
University of Edinburgh and Health Data Research UK	Andrew D Morris	
9	Angela Shore	Prof. Cardiovascular Science, PI
Peninsula Medical School	Kuni Aizawa	Postdoc
Exeter, UK	Claire Ball	Research nurse
	Nick Bellenger	Cardiologist
	Francesco Casanova	Associate Research Fellow Vascular medicine
	Tim Frayling	Prof. Genetics
	Phil Gates	Senior lecturer Cardiovascular science
	Kim Gooding	Postdoc Vascular medicine
	Andrew Hattersley	Prof. Molecular medicine
	Roland Ling	Consultant opthalmologist
	David Mawson	Research technician
	Robin Shandas	Prof. Bioengineering (Colorado)

	David Strain	Stroke physician, clinical lecturer
	Clare Thorn	Postdoc Vascular medicine
10	Ulf Smith	Prof. ; PI
University of Gothenburg	Ann Hammarstedt	Researcher Molecular and clinical medicine
Sweden	Hans Häring	Prof. University of Tübingen
	Oluf Pedersen	Prof. Steno Centre, Copenhagen
	Giorgio Sesti	Prof. Universtiy of Magna Graecia of Catanzaro
11	Per-Henrik Groop	Prof. Diabetes genetics; PI
	Emma Fagerholm	MSc; PhD student, genetics
Folkhälsan	Carol Forsblom	Clinical coordinator
Helsinki, Finland	Valma Harjutsalo	PhD; FinnDiane Co-PI
	Maikki Parkkonen	Laboratory manager
	Niina Sandholm	DSc(PhD); GWAS and bioinformatics, FinnDiane Co-PI
	Nina Tolonen	MD PhD
	Iiro Toppila	BSc, MSc; bioinformatician
	Erkka Valo	MSc; PhD student, bioinformatician
12	Veikko Salomaa	Prof. Epidemiology; PI; deputy leader WP2
The National Institute for Health and Welfare	Aki Havulinna	DSc. (tech), statistician
Helsinki, Finland	Kati Kristiansson	PhD
	Pia Okamo	THL press officer
	Tomi Peltola	PhD
	Markus Perola	Professor
	Arto Pietilä	Statistician
	Samuli Ripatti	Professor, Statistics
	Marketta Taimi	Research assistant
13	Seppo Ylä-Herttuala	Prof.; PI; WP4 leader
University of Eastern Finland	Mohan Babu	PhD student
Kuopio, Finland	Marike Dijkstra	PhD student
	Erika Gurzeler	PhD student
	Jenni Huusko	PhD student
	Ivana Kholová	Postdoc
	Markku Laakso	Prof.
	Mari Merentie	PhD student
	Marja Poikolainen	PA Prof Ylä-Herttuala

14	Mark McCarthy	Prof. Human type 2 diabetes; Oxford Centre for Diabetes, Endocrinology and Metabolism; Wellcome Trust Centre for Human Genetics; PI; deputy leader WP1
University of Oxford	Will Rayner	Database manager
UK	Neil Robertson	Informatics
	Natalie van Zuydam	Postdoc
15	Claudio Cobelli	Prof. ; PI; WP5 leader
University of Padova	Barbara Di Camillo	Assist. Prof.
Italy	Francesca Finotello	PhD student
	Francesco Sambo	Postdoctoral fellow
	Gianna Toffolo	Prof.
	Emanuele Trifoglio	PhD student
16	Riccardo Bellazzi	Prof. Bioengineering; PI; deputy leader WP5
	Nicola Barbarini	Postdoctoral fellow
University of Pavia	Mauro Bucalo	Software engineer
Italy	Christiana Larizza	Assist. Prof.
	Paolo Magni	Assoc. Prof.
	Alberto Malovini	Postdoctoral fellow
	Simone Marini	Postdoctoral fellow
	Francesca Mulas	Postdoctoral fellow
	Silvana Quaglini	Prof.
	Lucia Sacchi	Assist. Prof.
	Francesca Vitali	
17	Ele Ferrannini	Prof. Medicine; PI
	Beatrice Boldrini	Postdoctoral fellow
University of Pisa	Michaela Kozakova	Senior investigator Medical Pathophysiology
Italy	Andrea Mari	Senior researcher Biomedical engineering (ISIB-CNR, Padova)
	Carmela Morizzo	Biologist, Sonographer Cardiovascular ultrasound
	Lucrecia Mota	EGIR administrative office
	Andrea Natali	Assoc. Prof. Medicine
	Carlo Palombo	Assoc. Prof. Medicine; deputy leader WP3
	Elena Venturi	Researcher
	Mark Walker	Prof. Molecular diabetic medicine (Univ Newcastle-upon-Tyne)

18	Carlo Patrono	Prof.Pharmacology; PI
Catholic University of Rome	Francesca Pagliaccia	PhD student
Italy	Bianca Rocca	Assist. Prof. Pharmacology
19	Pirjo Nuutila	Prof. ; PI
University of Turku	Johanna Haukkala	PhD student
Finland	Juhani Knuuti	Prof. ; Director Turku PET Centre
	Anne Roivainen	Prof.
	Antti Saraste	Adj. Prof.
20	Paul McKeague	Prof. Genetic Epidemiology; PI
University of Edinburgh	Norma Brown	Research administrator, Public Health Services
Scotland	Marco Colombo	Bioinformaticist
21	Birgit Steckel-Hamann	Deputy coordinator; PI, Manager IMI, LRL
Eli Lilly	Krister Bokvist	Biostatistician
	Sudha Shankar	Diabetologist
	Melissa Thomas	Translational Science
22	Li-ming Gan	Prof.; Translational Science Director Cardiovascular Disease; PI, WP3 leader
AstraZeneca	Suvi Heinonen	PhD, Internal AZ postdoc, Bioscience
	Ann-Cathrine Jönsson-Rylander	PhD, Assoc. Prof., Team Leader Bioscience, WP4 leader
	Remi Momo	Postdoctoral fellow
	Volker Schnecke	Informatician Translational Science, WP5 leader
	Robert Unwin	Translational Science Director Diabetic Nephropathy
	Anna Walentinsson	Geneticist Translational Science
	Carl Whatling	Bioscientist
23	Everson Nogoceke	Pre-clinical and clinical aspects of metabolic and vascular disease; PI; WP2 leader
Roche	Gonzalo Durán Pacheco	Senior Research Statistician
	Ivan Formentini	Biomarker & Experimental Medicine Leader
	Thomas Schindler	Pre-clinical and clinical and clinical biomarkers
24	Piero Tortoli	Professor of Electronics
University of Florence	Luca Bassi	Postdoctoral fellow
	Enrico Boni	Postdoctoral fellow
	Alessandro Dallai	Postdoctoral fellow
	Francesco Guidi	Technician

	**Matteo Lenge**	**PhD student**
	**Riccardo Matera**	**PhD student**
	**Alessandro Ramalli**	**PhD student**
	**Stefano Ricci**	**Assist. Prof.**
	**Jacopo Viti**	**PhD student**
		-
**25**	**Bernd Jablonka**	**SAD internal IMI coordinator**
**Sanofi-aventis**	**Dan Crowther**	**Biomarker researcher**
	**Johan Gassenhuber**	**Biostatistician**
	**Sibylle Hess**	**Biomarker researcher**
	**Thomas Hübschle**	**Pharmacologist Diabetes**
	**Hans-Paul Juretschke**	**Imaging**
	**Hartmut Rütten**	**Head Translational Medicine**
	**Thorsten Sadowski**	**Pharmacologist Diabetes**
	**Paulus Wohlfart**	**Pharmacologist Diabetes**
		-
**26**	**Julia Brosnan**	**Biochemist, (pre)clinical research CVD, Pfizer US; WP2 leader**
**Pfizer**	**Valerie Clerin**	**Cardio-renal biologist, WP2**
	**Eric Fauman**	**Computational biologist**
	**Craig Hyde**	**Statistician**
	**Anders Malarstig**	**Human genetics, Pfizer Europé; WP1 leader**
	**Nick Pullen**	**Renal Disease Research Director**
	**Mera Tilley**	
	**Theresa Tuthill**	**Imaging specialist**
	**Ciara Vangjeli**	**Cardiovascular genetic epidemiologist, Pfizer Europe**
	**Daniel Ziemek**	**Computational biologist**

References

1. Webb TR, Erdmann J, Stirrups KE, Stitziel NO, Masca NGD, Jansen H, Kanoni S, Nelson CP, Ferrario PG, König IR, et al. Systematic Evaluation of Pleiotropy Identifies 6 Further Loci Associated With Coronary Artery Disease. J *Am Coll Cardiol.* 2017;69:823–836.
2. Voight BF, Kang HM, Ding J, Palmer CD, Sidore C, Chines PS, Burtt NP, Fuchsberger C, Li Y, Erdmann J, et al. The Metabochip, a Custom Genotyping Array for Genetic Studies of Metabolic, Cardiovascular, and Anthropometric Traits. *PLoS Genet.* 2012;8:e1002793.
3. Mägi R, Morris AP. GWAMA: software for genome-wide association meta-analysis. *BMC Bioinformatics.* 2010;11:288.
4. R Core Team. R: A Language and Environment for Statistical Computing 2013. R Foundation for Statistical Computing. 2019. Vienna, Austria.
5. Zheng J, Erzurumluoglu AM, Elsworth BL, Kemp JP, Howe L, Haycock PC, Hemani G, Tansey K, Laurin C, Pourcain BS, et al. LD Hub: A centralized database and web interface to perform LD score regression that maximizes the potential of summary level GWAS data for SNP heritability and genetic correlation analysis. *Bioinformatics.* 2017;33:272–279.
6. Shungin D, Winkler TW, Croteau-Chonka DC, Ferreira T, Locke AE, Mägi R, Strawbridge RJ, Pers TH, Fischer K, Justice AE, et al. New genetic loci link adipose and insulin biology to body fat distribution. *Nature* 2015;518:187–196.
7. Locke AE, Kahali B, Berndt SI, Justice AE, Pers TH, Day FR, Powell C, Vedantam S, Buchkovich ML, Yang J, et al. Genetic studies of body mass index yield new insights for obesity biology. *Nature.* 2015;518:197–206.
8. Thorleifsson G, Walters GB, Gudbjartsson DF, Steinthorsdottir V, Sulem P, Helgadottir A, Styrkarsdottir U, Gretarsdottir S, Thorlacius S, Jonsdottir I, et al. Genome-wide association yields new sequence variants at seven loci that associate with measures of obesity. *Nat Genet*. 2009;41:18–24.
9. Newton-Cheh C, Johnson T, Gateva V, Tobin MD, Bochud M, Coin L, Najjar SS, Zhao JH, Heath SC, Eyheramendy S, et al. Genome-wide association study identifies eight loci associated with blood pressure. *Nat Genet*. 2009;41:666–676.
10. Ehret GB, Munroe PB, Rice KM, Bochud M, Johnson AD, Chasman DI, Smith AV, Tobin MD, Verwoert GC, Hwang SJ, et al. Genetic variants in novel pathways influence blood pressure and cardiovascular disease risk. *Nature.* 2011;478:103–109.
11. Willer CJ, Sanna S, Jackson AU, Scuteri A, Bonnycastle LL, Clarke R, Heath SC, Timpson NJ, Najjar SS, Stringham HM, et al. Newly identified loci that influence lipid concentrations and risk of coronary artery disease. *Nat Genet*. 2008;40:161–169.

12. Mahajan A, Taliun D, Thurner M, Robertson NR, Torres JM, Rayner NW, Payne AJ, Steinthorsdottir V, Scott RA, Grarup N, et al. Fine-mapping type 2 diabetes loci to single-variant resolution using high-density imputation and islet-specific epigenome maps. *Nat Genet.* 2018;50:1505–1513.

13. Saxena R, Hivert M-F, Langenberg C, Tanaka T, Pankow JS, Vollenweider P, Lyssenko V, Bouatia-Naji N, Dupuis J, Jackson AU, et al. Genetic variation in *GIPR* influences the glucose and insulin responses to an oral glucose challenge. *Nat Genet.* 2010;42:142–148.

14. Manning AK, Hivert M-F, Scott RA, Grimsby JL, Bouatia-Naji N, Chen H, Rybin D, Liu CT, Bielak LF, Prokopenko I, et al. A genome-wide approach accounting for body mass index identifies genetic variants influencing fasting glycemic traits and insulin resistance. *Nat Genet.* 2012;44:659–669.

15. Soranzo N, Sanna S, Wheeler E, Gieger C, Radke D, Dupuis J, Bouatia-Naji N, Langenberg C, Prokopenko I, Stolerman E, et al. Common variants at 10 genomic loci influence hemoglobin A1C levels via glycemic and nonglycemic pathways. *Diabetes.* 2010;59:3229–3239.

16. Strawbridge RJ, Dupuis J, Prokopenko I, Barker A, Ahlqvist E, Rybin D, Petrie JR, Travers ME, Bouatia-Naji N, Dimas A S, et al. Genome-Wide Association Identifies Nine Common Variants Associated With Fasting Proinsulin Levels and Provides New Insights Into the Pathophysiology of Type 2 Diabetes. *Diabetes.* 2011;60:2624–2634.

17. Dupuis J, Langenberg C, Prokopenko I, Saxena R, Soranzo N, Jackson AU, Wheeler E, Glazer NL, Bouatia-Naji N, Gloyn AL, et al. New genetic loci implicated in fasting glucose homeostasis and their impact on type 2 diabetes risk. *Nat Genet.* 2010;42:105–116.

18. Lotta LA, Gulati P, Day FR, Payne F, Ongen H, van de Bunt M, Gaulton KJ, Eicher JD, Sharp SJ, Luan J, et al. Integrative genomic analysis implicates limited peripheral adipose storage capacity in the pathogenesis of human insulin resistance. *Nat Genet.* 2017;49:17–26.

19. Burgess S. R: A Language and Environment for Statistical Computing Package “MendelianRandomization” 2018.

20. Burgess S, Butterworth A, Thompson SG. Mendelian Randomization analysis with multiple genetic variants using summarized data. *Genet Epidemiol.* 2013;37:658–665.
