# Supplementary material for: Genetic Predisposition to Coronary Artery Disease in Type 2 Diabetes Mellitus
Source: Circ Genom Precis Med. 2020 Aug 13;13(6):e002769. doi: 10.1161/CIRCGEN.119.002769 (PMC7748049; doi:10.1161/CIRCGEN.119.002769)
Supplement: Supplementary file 3 [file hcg-13-e002769-s003.pdf]

Genetic Predisposition to Coronary Artery Disease in Type 2 diabetes

Running title: *van Zuydam et al.; Genetics of heart disease in type 2 diabetes*

Natalie R. van Zuydam, PhD[1-3]; Claes Ladenvall, PhD[4]; Benjamin F. Voight, PhD[5-7]; Rona J. Strawbridge, PhD[8]; Juan Fernandez Tajes, PhD[3]; N. William Rayner, PhD[2,3,9]; Neil R. Robertson, MSc[2,3]; Anubha Mahajan, PhD[2,3]; Efthymia Vlachopoulou, PhD[10]; Anuj Goel, MBBS, MSc[3,11]; Marcus E. Kleber, MD[12]; Christopher P. Nelson, PhD[13,14]; Lydia Coulter Kwee, PhD[15]; Tõnu Esko, PhD[16]; Evelin Mihailov, MSc[16]; Reedik Mägi, PhD[16]; Lili Milani, PhD[16]; Krista Fischer, PhD[16]; Stavroula Kanoni, PhD[17,18]; Jitender Kumar, PhD[19,20]; Ci Song, PhD[19,21-23]; Jaana A. Hartiala, PhD[24]; Nancy L. Pedersen, PhD[25]; Markus Perola, PhD[16,26,27]; Christian Gieger, PhD[28-30], Annette Peters, PhD[28,30,31]; Liming Qu, PhD[32]; Sara M. Willems, MD, PhD[33]; Alex S.F. Doney, MD, PhD[34]; Andrew D. Morris, MD, PhD[35,36]; Yan Zheng, PhD[37,38]; Giorgio Sesti, MD[39]; Frank B. Hu, MD, PhD[37,40,41]; Lu Qi, PhD[37,42]; Markku Laakso, PhD[43,44]; Unnur Thorsteinsdottir, PhD[45,46]; Harald Grallert, PhD[28,29,47,48]; Cornelia van Duijn, PhD[33]; Muredach P. Reilly PhD[32]; Erik Ingelsson, PhD[19,49-51]; Panos Deloukas, PhD[18,52]; Sek Kathiresan, MD[53-55]; Andres Metspalu, PhD[16,56]; Svati H. Shah, MD, MS, MHS[15,57]; Juha Sinisalo, MD, PhD[58]; Veikko Salomaa, MD, PhD[26]; Anders Hamsten, PhD[8]; Nilesh J. Samani, MD, PhD[13,14]; Winfried Maerz, MD, PhD[12,59,60]; Stanley L. Hazen, MD, PhD[61]; Hugh Watkins, MD, PhD[3,11]; Danish Saleheen, PhD[62,63]; SUMMIT Steering Committee; CARDIOGRAMplusC4D Steering Committee; Andrew P. Morris, PhD[3,64,65]; Helen M. Colhoun, MD, PhD[66,67]; Leif Groop, MD, PhD[4,68]; Mark I. McCarthy, MD, PhD[2,3,69,70]; Colin N.A. Palmer, PhD[1]

American Heart Association.

[1]Pat Macpherson Ctr for Pharmacogenetics & Pharmacogenomics, Cardiovascular & Diabetes Medicine, School of Medicine, [34]Division of Molecular & Clinical Medicine, School of Medicine, Univ of Dundee, Dundee; [2]Oxford Ctr for Diabetes, Endocrinology & Metabolism, Radcliffe Dept of Medicine, [3]Wellcome Ctr for Human Genetics, [11]Division of Cardiovascular Medicine, Radcliffe Dept of Medicine, Univ of Oxford, Oxford, UK; [4]Dept of Clinical Sciences, Diabetes & Endocrinology, Lund Univ Diabetes Ctr, Malmö, Sweden; [5]Dept of Systems Pharmacology & Translational Therapeutics, [6]Dept of Genetics, [7]Inst for Translational Medicine & Therapeutics, [32]Cardiovascular Inst, Univ of Pennsylvania, Perelman School of Medicine, Philadelphia, PA; [8]Cardiovascular Medicine Unit, Dept of Medicine Solna, Ctr for Molecular Medicine, Karolinska Institutet, Karolinska Univ Hospital Solna, Solna, Stockholm, Sweden; [9]Dept of Human Genetics, Wellcome Trust Sanger Inst, Hinxton, UK; [10]Transplantation Laboratory, Haartman Inst, [27]Rsrch Program for Clinical & Molecular Metabolism, Faculty of Medicine, Univ of Helsinki, Helsinki, Finland; [12]Vth Dept of Medicine (Nephrology, Hypertensiology, Rheumatology, Endocrinology, Diabetology), Medical Faculty Mannheim, Heidelberg Univ, Mannheim, Germany; [13]Dept of Cardiovascular Sciences, Univ of Leicester & [14]NIHR Leicester Biomedical Rsrch Ctr, Glenfield Hospital, Leicester, UK; [57]Division of Cardiology, Dept of Medicine, Duke Univ Medical Ctr, [15]Duke Molecular Physiology Inst, Duke Univ, Durham, NC; [16]Estonian Genome Ctr, [56]Inst of Cell & Molecular Biology, Univ of Tartu, Tartu, Estonia; [17]Ctr for Genomic Health, [18]William Harvey Rsrch Inst, Barts & the London Medical School, Queen Mary Univ of London, London, UK; [19]Dept of Medical Sciences, Molecular Epidemiology & Science for Life Laboratory, [21]Dept of Immunology, Genetics and Pathology, Medical Genetics & Genomics, Uppsala Univ, Uppsala, Sweden; [20]Ctr for Computational Biology & Bioinformatics, Amity Inst of Biotechnology, Amity Univ Uttar Pradesh, Noida, India; [22]Framingham Heart Study; [23]Population Sciences Branch, National Heart, Lung & Blood Inst, National Inst of Health, Framingham, MA; [24]Dept of Preventive Medicine, Keck School of Medicine, Univ of Southern California, Los Angeles, CA; [25]Dept of Medical Epidemiology & Biostatistics, Karolinska Institutet, Stockholm, Sweden; [26]National Institute for Health and Welfare, Helsinki, Finland; [28]German Ctr for Diabetes Rsrch (DZD), München-Neuherberg; [29]Clinical Cooperation Group Type 2 Diabetes, [30]German Rsrch Ctr for Environmental Health & Inst of Genetic Epidemiology, [47]Rsrch Unit of Molecular Epidemiology, Inst of Epidemiology, [48]Clinical Cooperation Group Nutrigenomics & Type 2 Diabetes, Helmholtz Zentrum München, Neuherberg; [31]DZHK (German Ctr for Cardiovascular Research), partner site Munich Heart Alliance, Munich, Germany; [33]Dept of Epidemiology, Erasmus Medical Ctr, Rotterdam, the Netherlands; [35]The Usher Inst of Population Health Sciences & Informatics, [66]MRC Inst of Genetics & Molecular Medicine, Univ of Edinburgh, Edinburgh; [36]Health Data Research UK, London, UK; [37]Dept of Nutrition, [40]Dept of Epidemiology, Harvard School of Public Health, Boston, MA; [38]Ministry of Education Key Laboratory of Contemporary Anthropology, School of Life Sciences, Fudan Univ, Shanghai, China; [39]Univ "Magna Graecia" of Catanzaro, Italy; [41]Channing Division of Network Medicine, Dept of Medicine, Brigham and Women's Hospital & Harvard Medical School, Boston, MA; [42]Dept of Epidemiology, School of Public Health & Tropical Medicine, Tulane Univ, New Orleans, LA; [43]Faculty of Health Sciences, Inst of Clinical Medicine, Internal Medicine, Univ of Eastern Finland, [44]Kuopio University Hospital, Kuopio, Finland; [45]Faculty of Medicine, Univ of Iceland, [46]deCODE Genetics, Reykjavik, Iceland; [49]Dept of Medicine, Division of Cardiovascular Medicine, Stanford Univ School of Medicine; [50]Stanford Cardiovascular Inst, [51]Stanford Diabetes Rsrch Ctr, Stanford Univ, Stanford, CA; [52]Princess Al-Jawhara Al-Brahim Ctr of Excellence in Rsrch of Hereditary Disorders (PACER-HD), King Abdulaziz Univ, Jeddah, Saudi Arabia; [53]Broad Inst of MIT & Harvard, Cambridge; [54]Cardiology Division, Cte for Human Genetic Rsrch, [55]Cardiovascular Rsrch Ctr, Massachusetts General Hospital & Harvard Medical School, Boston, MA; [58]Heart & Lung Ctr, Helsinki Univ Hospital, [68]Institute for Molecular Medicine Finland (FIMM), Helsinki Univ, Helsinki, Finland; [59]Synlab Academy, Synlab Holding Deutschland GmbH, Mannheim, Germany; [60]Clinical Inst of Medical & Chemical Laboratory Diagnostics, Medical Univ of Graz, Graz, Austria; [61]Lerner Research Inst, Heart & Vascular Institute, Cleveland Clinic, Cleveland, OH; [62]Dept of Biostatistics & Epidemiology, Univ of Pennsylvania, Philadelphia, PA; [63]Ctr for Non-Communicable Diseases, Karachi, Pakistan; [64]Dept of Biostatistics, Univ of Liverpool, Liverpool; [65]Division of Musculoskeletal & Dermatological Sciences, Univ of Manchester, Manchester; [67]Public Health, NHS Fife, Kirkcaldy; [69]Oxford NIHR Biomedical Rsrch Ctr, Oxford Univ Hospitals NHS Foundation Trust, John Radcliffe Hospital, Oxford, UK; [70]Currently: Genentech, San Francisco, CA

A full list of members & affiliations of the SUMMIT and CARDIoGRAM*plus*C4D appears in the supplementary material

Correspondence:

Natalie R. van Zuydam, PhD

Uppsala University

Department of Immunology, Genetics and Pathology

Science for Life Laboratory (SciLifeLab)

Box 815, 75 108 Uppsala

Sweden

Tel: +46 70 489 6605

E-mail: natalie.vanzuydam@igp.uu.se

Journal Subject Terms: Genetic, Association Studies; Genetics; Diabetes, Type 2; Coronary Artery Disease

Abstract:

Background - Coronary artery disease (CAD) is accelerated in subjects with type 2 diabetes (T2D).

Methods - To test whether this reflects differential genetic influences on CAD-risk in subjects with T2D, we performed a systematic assessment of genetic overlap between CAD and T2D in 66,643 subjects (27,708 with CAD and 24,259 with T2D). Variants showing apparent association with CAD in stratified analyses and/or evidence of interaction were evaluated in a further 117,787 subjects (16,694 with CAD and 11,537 with T2D).

Results - None of the previously characterised CAD loci was found to have specific effects on CAD in T2D individuals and a genome-wide interaction analysis found no new variants for CAD that could be considered T2D specific. When we considered the overall genetic correlations between CAD and its risk factors, we found no substantial differences in these relationships by T2D background.

Conclusions - This study found no evidence that the genetic architecture of CAD differs in those with T2D compared to those without T2D.

Circulation: Genomic and Precision Medicine

Key words: coronary artery disease; type 2 diabetes mellitus; genetic association

Nonstandard Abbreviations and Acronyms

Coronary artery disease - CAD

Type 2 diabetes - T2D

Coronary ARtery DIsease Genome wide Replication and Meta-analysis (CARDIoGRAM) plus The Coronary Artery Disease (C4D) Genetics - CARDIoGRAM*plus*C4D

European Network for Genetic and Genomic Epidemiology - ENGAGE

SUrrogate markers for Micro- and Macro-vascular hard endpoints for Innovative diabetes Tools - SUMMIT

Genome-wide association study - GWAS

The Nurses' Health study - NHS

The Metabolic Syndrome in Men – METSIM

The Health Professionals Follow-Up Study - HPFS

Odds ratio - OR

Effect allele frequency - EAF

Genetic risk scores - GRS

Low-density lipoprotein cholesterol - LDL-C

Body mass index - BMI

Systolic blood pressure - SBP

American Heart Association.
Circulation: Genomic and Precision Medicine

Introduction

There is considerable variation in the presentation, severity, and pathology of coronary artery disease (CAD) between subjects with type 2 diabetes (T2D) and those with no history of diabetes. Subjects with T2D have more extensive and severe atherosclerosis, suffer more silent infarcts and are more prone to thrombosis than subjects without diabetes.[1–3] The mechanisms by which T2D accelerates CAD are poorly understood. In principle, the acceleration of CAD in T2D may be attributed to features that jointly predispose subjects to T2D and CAD, and/or to

factors intrinsic to the T2D state that increase the risk of CAD, such as hyperglycaemia, insulin resistance and chronic inflammation.[4]

Predisposition to both CAD and T2D has a substantial genetic component (with ~163 CAD-risk and ~403 for T2D association signals identified to date in subjects of European Ancestry)[5,6] and Mendelian randomisation studies support a causal role for T2D in the development of CAD.[7–9] A Mendelian randomisation study found that the average CAD risk per T2D allele was lower than expected (for the 44 T2D associated variants assessed) compared to the increased risk of CAD attributed to T2D by epidemiological studies.[7] This indicated that the T2D associated variants did not account for all the risk of CAD observed in subjects with T2D. Few variants have been associated with both CAD and T2D: a variant near *IRS1* was associated with both diseases at genome-wide significance ($p \leq 5 \times 10^{-8}$) and eight other loci at a lower significance level.[9] Given that there are few variants jointly associated with CAD and T2D it is unsurprising that there is sparse evidence for overlapping pathways contributing to both diseases.[10]

Circulation: Genomic and Precision Medicine

A recent study conducted in the UK Biobank found no evidence of differential effects of CAD-risk variants by T2D status. However, in this study, the sample size was relatively small (3,968 CAD cases and 11,698 controls).[11] Another study found that a genetic risk score constructed from known CAD loci was associated with CAD in subjects with T2D, indicating that variants identified in the general population were predictive of CAD in the context of T2D.[12] What has not been systematically addressed in a large sample, is whether there is a quantitative or qualitative difference in the pattern of loci influencing risk of CAD amongst subjects with T2D when compared to those without the condition.

We conducted a comprehensive investigation of genetic differences in the determinants of CAD between subjects with and without T2D in a large sample. The discovery meta-analysis included

66,643 subjects (of whom 27,708 had CAD and 24,259 had T2D) and we sought replication for a subset of variants in a further 117,787 samples (16,694 with CAD; 11,537 subjects with T2D).

Methods

An overview of the study design is illustrated in figure 1 and the methods are provided in the supplementary material. The summary statistics have been made available via figshare (10.6084/m9.figshare.7811639). This study made use of data generated from individual studies for which the relevant institutional review board approval had been obtained and all participants consented to inclusion in individual studies.

American Heart Association.

Results

Identification of CAD cases, CAD controls and subjects with diabetes

Circulation: Genomic and Precision Medicine

This study was performed using full summary statistics from CAD case-control analyses performed separately in subjects with T2D and subjects with no history of diabetes. The discovery meta-analyses included 27,708 CAD cases (of whom 10,014 had T2D) and 38,935 subjects with no history of CAD (14,245 with T2D) from 23 studies of European descent and one study of South Asian descent, assembled from the CARDIoGRAM*plus*C4D, ENGAGE and SUMMIT consortia (Figure 1 and Supplementary Tables 1 and 2). Replication of selected signals was sought in an independent sample of 16,694 CAD cases (3,706 with T2D) and 101,093 controls with no history of CAD (7,831 with T2D) from four studies of European descent with existing GWAS from deCODE, the Nurses' Health study (NHS), the Metabolic Syndrome in Men (METSIM) and the Health Professionals Follow-Up Study (HPFS) (Supplementary Tables 3 and 4). None of the studies contained overlapping samples.

Main effects of variants on CAD

We first set out to identify variants that were associated with CAD in the complete sample set. We performed two meta-analyses, the first compared CAD cases to controls without reference to T2D status, whilst the second repeated the analysis adjusted for T2D status. In both analyses, we confirmed many of the previously reported CAD associated loci at genome-wide significance ($p \leq 5\times10^{-8}$) including *SORT1/CELSR2*, *WDR12*, *PHACTR1*, *TCF21*, 9p21.3, *CXCL12* and *ADAMTS7*. We selected 142 variants that achieved $p \leq 5\times10^{-4}$ in either the unadjusted or the T2D-adjusted analyses for replication analyses.

We had access to full summary statistics for the discovery analysis and but not from the replication cohorts. We requested summary statistics for selected variants from replication cohorts. Thus, we performed a joint analysis of the estimates from the discovery analyses and replication analyses. In the joint analysis, we expanded the set of known CAD loci detected in this meta-analysis from 7 to 13 reaching genome-wide significance in our dataset (Figure 2A, 2B and Supplementary Table 5). For published CAD variants, the risk allele identified in this meta-analysis was the same as the published risk allele for variants associated with CAD $p \leq 1\times10^{-3}$ (Supplementary Figure 2 and Supplementary Table 5).[5] This reflects, in part, an overlap of samples included in these various analyses (Supplementary Figure 2 and Supplementary Table 5).

American Heart Association.
Circulation: Genomic and Precision Medicine

Stratified analysis

The second approach we employed to identify any loci at which CAD-risk effects ($p \leq 5\times10^{-8}$) were influenced by the presence or absence of T2D, involved a T2D-stratified meta-analysis of CAD-risk. In the discovery phase of this stratified analysis, three known CAD loci reached genome-wide significance: *ADAMTS7* in subjects with T2D and 9p21.3 and *PHACTR1* in the

analysis of subjects without diabetes (Supplementary Table 5). The allelic effects and association signals at the previously reported CAD-loci did not show any systematic difference according to T2D background (Supplementary Figure 1).

We selected 230 lead variants for replication from the T2D-only analysis and 175 lead variants from the analysis of subjects without diabetes for replication based on a stratum-specific CAD association of p≤1×10-4. In the joint analysis (discovery and replication), we found no novel CAD-risk signals in either stratum (Figure 2 C, D and Supplementary Table 5). Three loci were associated with CAD in subjects with T2D and these overlapped loci associated with CAD in subjects without diabetes (Figure 2). The different number of loci associated with CAD by T2D background reflects a difference in power (i.e. sample size) to detect associations rather than a systematic difference by T2D background.

American Heart Association.

Interaction analysis

Circulation: Genomic and Precision Medicine

In a complementary analysis to the stratified analysis, we performed a T2D interaction analysis (see Supplementary Methods), to identify variants that interacted with T2D status to modify the risk of CAD. We calculated the interaction *p* values based on summary statistics from the T2D stratified analyses of CAD and not from a meta-analysis of interaction terms. We adopted this approach to maximise the number of samples used to estimate interactive effects (see Supplementary Methods). The interaction analysis was performed by comparing the allelic effects (on the log odds scale) on CAD risk for each variant between T2D strata. The allelic effects and their associated standard errors for CAD risk estimated in T2D stratified meta-analyses were compared using GWAMA v2.1.[13] The smaller the $p_{interaction}$ the larger difference in allelic effects on CAD risk by T2D status.

The top interaction in the discovery analysis was represented by rs712755, near *GRM7*

($p_{interaction}$=4.6×10^{-7}). This variant had opposing effects on CAD risk dependent on T2D context (EAF= 0.71, OR_{T2D} R, 0.82[0.74-0.90], $OR_{NoDiabetes}$, 1.14 [1.06-1.23]).

We sought replication for 175 loci, including *GRM7,* with at least modest evidence of interaction with T2D status ($p_{interaction} \leq 1\times10^{-4}$). We performed a joint interaction meta-analysis of the discovery and replication data and defined replication as a combined (discovery+replication) $p_{interaction}<2.9\times10^{-4}$ (0.05/175; that corrects for the number of loci selected for replication), and a joint $p_{interaction}$< discovery $p_{interaction}$. The latter indicates directionally consistent allelic effects by T2D stratum in the discovery and replication stages.

The interaction at *GRM7*, represented by rs712755, did not replicate (replication $p_{interaction}$=0.36) and none of the other 174 loci met the criteria for replication. Overall, there was no evidence for loci that interacted with T2D status to modify the risk of CAD based on this interaction analysis.

Circulation: Genomic and Precision Medicine

We also examined the known CAD loci for evidence of interaction. Of the 163 known variants for CAD, 161 were present in our data. We applied a Bonferroni correction of $p_{interaction}\leq3.1\times10^{-4}$ (0.05/161; correcting for the number of known CAD loci). None of the established CAD loci interacted with T2D status to modify the risk of CAD (Supplementary Table 5). A variant located near *GLUL* (rs10911021), had been associated with CAD in subjects with T2D.[14] In the current study, rs10911021 showed no association with CAD in subjects with T2D (p=0.54) and had no evidence of interaction with T2D status ($p_{interaction}$=0.46; Supplementary Figure 3).

Power to detect interactions

A substantial challenge in detecting loci that interact with T2D to modify the risk of CAD is sufficient sample size. Even in this large discovery sample of 66,643 subjects (27,708 with

CAD), we had <80% power to detect interactions with at least a 20% difference in allelic odds between strata (i.e. $OR_{NoDiabetes}$=1.00 vs OR_{T2D}=1.20) for MAF>10% at □=1×10^{-4} (the threshold for replication selection in the interaction analysis) (see Supplementary Methods). This was only for interactions where there were opposite allelic effects in strata or where there was a null allelic effect on CAD in one stratum (i.e. $OR_{NoDiabetes}$=1.00) and a large (i.e. OR_{T2D}=1.20) allelic effect on CAD in the other stratum (Supplementary Figure 2A and 2B). We had little power to detect interactions where allelic effects on CAD were in the same direction in both strata (see Supplementary Methods; Supplementary Figure 2C). In the replication sample of 117,787 samples (16,694 with CAD) at an α=0.05 we observed similar patterns of power to detect associations with opposing effects by stratum. Thus, we would be unlikely (in this sample size) be able to detect smaller interaction effects and/or those involving rare alleles.

American Heart Association.
Circulation: Genomic and Precision Medicine

Genetic overlap with risk factors

We have comprehensively interrogated variants for association with CAD in the context of T2D, but not risk factors of both T2D and CAD. There may be a different effect of these risk factors on CAD by T2D context which may explain some of the increased risk of CAD in subjects with T2D. First, we performed genetic correlation analyses using LDHub to estimate the overall genetic correlation (based on all variants) between risk factors and CAD separately by T2D background.[15] Subsequently, a heterogeneity test was performed on the risk factor genetic correlation estimates with CAD by T2D background to identify risk factors that may have a variable correlation with CAD based on T2D background. Overall, we found no difference in the genetic correlation between 106 risk factors and CAD by T2D status (Supplementary Figure 4; Supplementary Table 6).

To investigate this further but only in a subset of variants associated with risk factors at genome-wide significance ($p \leq 5\times10^{-8}$), we constructed weighted genetic risk scores (GRS) for seventeen traits related to obesity,[16–18] hypertension,[19] lipids,[20] diabetes,[6,21]glycaemic traits and insulin resistance.[22–28] These GRSs included between 10 and 403 SNPs for each phenotype. We tested these for CAD association in the T2D unadjusted ("main") analysis, as well as in the T2D-stratified analyses, where we performed a test for heterogeneity for different effects on CAD by T2D background (see Supplementary Methods). We adopted a significance threshold of $p \leq 2.9\times10^{-3}$ that accounted for the 17 GRS, but not for the multiple CAD associations performed. Genetic risk scores for low-density lipoprotein cholesterol (LDL-C), body mass index (BMI) and systolic blood pressure (SBP) were associated with CAD irrespective of T2D background (Supplementary Figure 5; Supplementary Table 7). Collectively, these analyses provide no evidence to support T2D-stratified differences in CAD-risk as conveyed by variants influencing phenotypes known to contribute to CAD development.

American Heart Association.
Circulation: Genomic and Precision Medicine

Discussion

There is a well-established causal role for T2D in increased risk of CAD. However, this increased risk could not be explained by differences in genetic architecture of CAD between individuals with and without diabetes. We found no difference in the effects of known CAD loci on the risk of CAD by T2D status. We also found no variants of large effect specifically associated with CAD in the context of T2D. We also found no differences in the effects of risk factors on CAD by T2D background based on analyses that used the genetic variation contributing to these risk factors. Indicating that the genetic variants associated with these risk factors do not have a differential effect on CAD risk by T2D background.

There are a number of factors that will influence the power to detective genuine interactive effects. Identification of interactive effects requires a large sample size particularly when conducting a genome-wide interaction analysis.[29] Even in this study that included 66, 643 subjects (considerably larger than previous efforts), we were underpowered to identify variants with small differences in effect on CAD risk by T2D status. If interaction effects do exist, these effects are likely to be modest and only detectable in a much larger sample size.

The accuracy of the phenotype definition will also affect the power to detect interactive effects. Diagnosis of T2D is often contemporaneous to CAD diagnosis and may not reflect the actual onset of diabetes. We are uncertain of the stage of T2D development when risk of CAD begins to increase. There is evidence of increased vascular risk before the onset of clinically diagnosed T2D.[30] Taking this variability into account, we defined CAD cases with T2D as those that had a diagnosis of T2D up to 5 years after a CAD event with no minimum duration of diabetes. This also allowed us to increase the sample size by including cross-sectional studies for which information on the duration of diabetes may not be available. We were unable to account for the attenuation of genetic effects due to the misclassification of subjects who may develop CAD and or T2D outside of the study observation period.

This study shows that difference in risk of CAD between subjects with and without T2D cannot be explained by variants of large effect or differences in the genetic variation contributing to known risk factors of either T2D or CAD. There are several other mechanisms, outside the scope of the current study, that could explain some of the increased risk of CAD in subjects with T2D. There could be epigenetic changes induce by some feature of the T2D state. For example, hyperglycaemia has been shown to cause epigenetic changes altering gene expression in vascular cells leading to endothelial dysfunction, a hallmark of atherosclerosis.[31] Although the evidence

Circulation: Genomic and Precision Medicine

for overlapping pathways between CAD and T2D is sparse, treatment of one disease can increase the risk of the other. Statins known to reduce the risk of CAD, have been shown to increase the risk of T2D; whilst some thialidazones, used to treat insulin resistance in subjects with T2D, increase the risk of CAD.[32] It is likely that the T2D state perturbs or exacerbates some common atherosclerotic processes rather than through T2D background specific genes/pathways to increase the risk of CAD in subjects with T2D.

Sources of Funding: This work was supported by the European Union Framework Programme 7 (FP7/2007-2013) for the Innovative Medicine Initiative (IMI) under grant agreement n° IMI/115006 (the SUMMIT consortium); Aarno Koskelo Foundation; Academy of Finland (#263401; #2676882); American Heart Association (13SDG14330006); AstraZeneca; AtheroSysMed (Systems medicine of coronary heart disease and stroke); British Heart Foundation Centre of Research Excellence at Oxford; ERC269045-Gene Target T2D grant; Estonian Research Council (IUT20-60, PUT1660 and PUT1665P); Estonian Center of Genomics/Roadmap II (project No. 2014-2020.4.01.16-0125); European Union (#692145; #633589; #313010; LSHM-CT-2007-037273; #201668; 2014-2020.4.01.15-0012;QLG1-CT-2002-00896; EU/QLRT-2001-01254; QLG2-CT-2002-01254 HEALTH-F2-2013-601456); Finnish Foundation for Cardiovascular research; Gentransmed - Centre of Excellence for Genomics and Translational Medicine; German Ministry of Education and Research (#01ZX1313A-K); Helsinki University Central Hospital special government funds (EVO #TYH7215, #TKK2012005, #TYH2012209, #TYH2014312); Juvenile Diabetes Research Foundation (JDRF, 2-SRA-2014-276-Q-R); National Institute of Diabetes and Digestive and Kidney diseases (NIDDK, 5R01DK106236; U01-DK066134; U01-DK105535; R01DK101478); National Heart, Lung and Blood Institute (NLHBI, R01HL103866); National Institute for Health Research (NIHR); RiskyCAD (305739); Sigrid Juselius Foundation; Finnish Academy (#269517); Finnish Foundation for Cardiovascular Research; Foundation for Strategic Research and Stockholm County Council (560283; 592229); Juho Vainio Foundation; Knut and Alice Wallenberg Foundation; Ministry for Higher Education; Strategic Cardiovascular and Diabetes Programmes of Karolinska Institutet and Stockholm County Council; Swedish Foundation for

American Heart Association.
Circulation: Genomic and Precision Medicine

Strategic Research (SSF; ICA08-0047); Swedish Heart-Lung Foundation; Swedish Research Council (project 8691; 2015-02558; 2016-00598; M-2005-1112 and 2009-2298); Torsten and Ragnar Söderberg Foundation; W.W. Smith Charitable Trust (H1201); Wellcome Trust Institutional strategic support fund; Yrjö Jahnsson Foundation.

Appendix:

CARDIoGRAMplusC4D: John Danesh, Jeanette Erdmann, Dongfeng Gu, Jaspal S Kooner, Robert Roberts, Heribert Schunkert, Themistocles L Assimes, Stefan Blankenberg, Bernhard O Boehm, John C Chambers, Robert Clarke, Rory Collins, George Dedoussis, Paul W Franks, G Kees Hovingh, Bong-Jo Kim, Terho Lehtimäki, Winfried März, Ruth McPherson, Markku S Nieminen, Christopher O'Donnell, Samuli Ripatti, Manjinder S Sandhu, Stefan Schreiber, Agneta Siegbahn, Cristen J Willer, Pierre A Zalloua

SUMMIT: Michael Mark, Timo Kanninen, Barbara Thorand, Giuseppe Remuzzi, David Dunger, Angela Shore, Ulf Smith, Per-Henrik Groop, Veikko Salomaa, Seppo Ylä-Herttuala, Claudio Cobelli, Riccardo Bellazzi, Ele Ferrannini, Carlo Patrono, Pirjo Nuutila, Paul McKeague, Birgit Steckel-Hamann, Li-ming Gan, Everson Nogoceke, Piero Tortoli, Bernd Jablonka, Mary-Julia Brosnan

American Heart Association.
Circulation: Genomic and Precision Medicine

Disclosures: Authors have disclosed possible conflicts of interest and have confirmed that these are unrelated to the work described in this manuscript. Erik Ingelsson is a scientific advisor for Precision Wellness. Veikko Salomaa has consulted for Novo Nordisk and Sanofi, and has ongoing research collaboration with Bayer (all unrelated to the present study). Dr W.M. März reports employment with Synlab Holding Deutschland GmbH and has received grants and/or personal fees from: Abott Diagnostics; Aegerion Pharmaceuticals; AMGEN; AstraZeneca; BASF; Danone Research; MSD; Sanofi; Siemens Diagnostics; and Synageva. Professor HM Colhoun receives research support and honoraria from and is also a member of the advisory panels and speaker’s bureaus for Sanofi Aventis, Regeneron, and Eli Lilly. Professor Colhoun has been a member of DSMB of the Advisory Panel for the CANTOS Trial (Novartis Pharmaceuticals). Professor HM Colhoun also receives or has recently received non-binding research support from Roche Pharmaceuticals, Pfizer Inc., Boehringer Ingelheim and AstraZeneca. Professor HM Colhoun is a shareholder of Roche Pharmaceuticals and Bayer.

MMcC has served on advisory panels for Pfizer, NovoNordisk and Zoe Global, has received honoraria from Merck, Pfizer, Novo Nordisk and Eli Lilly, and research funding from Abbvie, AstraZeneca, Boehringer Ingelheim, Eli Lilly, Janssen, Merck, NovoNordisk, Pfizer, Roche, Sanofi Aventis, Servier, and Takeda. As of June 2019, MMcC is an employee of Genentech, and a holder of Roche stock. As of September 2019, N.R.v.Z is an employee of AstraZeneca. As of 2016, Efthymia Vlachopoulou is an employee of Medpace. The following authors have indicated no conflicts of interest: Claes Ladenvall, Benjamin F Voight PhD, Rona J Strawbridge PhD, Juan Fernandez Tajes, N William Rayner, Neil R Robertson, Anubha Mahajan, Anuj Goel PhD, Marcus E Kleber, Christopher P Nelson, Lydia Coulter Kwee, Tõnu Esko, Evelin Mihailov, Reedik Mägi, Lili Milani, Krista Fischer, Stavroula Kanoni, Jitender Kumar, Ci Song, Jaana A Hartiala, Nancy L Pedersen, Markus Perola, Christian Gieger, Annette Peters, Liming Qu, Sara M Willems, Alex S F Doney, Andrew D Morris, Yan Zheng, Giorgio Sesti, Frank B Hu, Lu Qi, Markku Laakso, Unnur Thorsteinsdottir, Harald Grallert, Cornelia van Duijn, Muredach P Reilly, Panos Deloukas, Sek Kathiresan, Andres Metspalu, Svati Shah, Juha Sinisalo, Anders Hamsten, Nilesh J Samani, Stanley L Hazen, Hugh Watkins, Danish Saleheen, Andrew P Morris, Leif Groop, Colin N A Palmer.

American Heart Association.
Circulation: Genomic and Precision Medicine

References:

1. Waller BF, Palumbo PJ, Lie JT, Roberts WC. Status of the coronary arteries at necropsy in diabetes mellitus with onset after age 30 years. Analysis of 229 diabetic patients with and without clinical evidence of coronary heart disease and comparison to 183 control subjects. *Am J Med.* 1980;69:498–506.

2. Koistinen MJ. Prevalence of asymptomatic myocardial ischaemia in diabetic subjects. *Br Med J.* 1990;301:92–95.

3. Carr ME. Diabetes mellitus: a hypercoagulable state. *J Diabetes Complications.* 2001;15:44–54.

4. El-Osta A, Brasacchio D, Yao D, Pocai A, Jones PL, Roeder RG, Cooper ME, Brownlee M. Transient high glucose causes persistent epigenetic changes and altered gene expression during subsequent normoglycemia. *J Exp Med.* 2008;205:2409–2417.

5. Erdmann J, Kessler T, Munoz Venegas L, Schunkert H. A decade of genome-wide association studies for coronary artery disease: The challenges ahead. *Cardiovasc Res.* 2018;114:1241–1257.

6. Mahajan A, Taliun D, Thurner M, Robertson NR, Torres JM, Rayner NW, Payne AJ, Steinthorsdottir V, Scott RA, Grarup N, et al. Fine-mapping type 2 diabetes loci to single-variant resolution using high-density imputation and islet-specific epigenome maps. *Nat Genet.* 2018;50:1505–1513.

7. Jansen H, Loley C, Lieb W, Pencina MJ, Nelson CP, Kathiresan S, Peloso GM, Voight BF, Reilly MP, Assimes TL, et al. Genetic variants primarily associated with type 2 diabetes are related to coronary artery disease risk. *Atherosclerosis.* 2015;241:419–426.

8. Ross S, Gerstein HC, Eikelboom J, Anand SS, Yusuf S, Paré G. Mendelian randomization analysis supports the causal role of dysglycaemia and diabetes in the risk of coronary artery disease. *Eur Heart J.* 2015;36:1454–1462.

9. Zhao W, Rasheed A, Tikkanen E, Lee J-J, Butterworth AS, Howson JMM, Assimes TL, Chowdhury, R, Orho-Melander M, Damrauer S, et al. Identification of new susceptibility loci for type 2 diabetes and shared etiological pathways with coronary heart disease. *Nat Genet.* 2017;49:1450–1457.

10. Chan KHK, Huang Y-T, Meng Q, Wu C, Reiner A, Sobel EM, Tinker L, Lusis AJ, Yang X, Liu, S. Shared Molecular Pathways and Gene Networks for Cardiovascular Disease and Type 2 Diabetes Mellitus in Women Across Diverse Ethnicities. *Circ Cardiovasc Genet.* 2014;7:911–919.

11. Fall T, Gustafsson S, Orho-Melander M, Ingelsson E. Genome-wide association study of coronary artery disease among individuals with diabetes: the UK Biobank. *Diabetologia.* 2018;61:2174–2179.

American Heart Association.
Circulation: Genomic and Precision Medicine

12. Morieri ML, Gao H, Pigeyre M, Shah HS, Sjaarda J, Mendonca C, Hastings T, Buranasupkajorn P, Motsinger-Reif AA, Rotroff DM, et al. Genetic tools for coronary risk assessment in type 2 diabetes: A cohort study from the ACCORD clinical trial. *Diabetes Care.* 2018;41:2404–2413.

13. Mägi R, Morris AP. GWAMA: software for genome-wide association meta-analysis. *BMC Bioinformatics.* 2010;11:288.

14. Qi L, Qi Q, Prudente S, Mendonca C, Andreozzi F, Di Pietro N, Sturma M, Novelli V, Mannino GC, Formoso G, et al. Association between a genetic variant related to glutamic acid metabolism and coronary heart disease in individuals with type 2 diabetes. *JAMA.* 2013;310:821–828.

15. Zheng J, Erzurumluoglu AM, Elsworth BL, Kemp JP, Howe L, Haycock PC, Hemani G, Tansey K, Laurin C, Pourcain BS, et al. LD Hub: A centralized database and web interface to perform LD score regression that maximizes the potential of summary level GWAS data for SNP heritability and genetic correlation analysis. *Bioinformatics.* 2017;33:272–279.

16. Locke AE, Kahali B, Berndt SI, Justice AE, Pers TH, Day FR, Powell C, Vedantam S, Buchkovich ML, Yang J, et al. Genetic studies of body mass index yield new insights for obesity biology. *Nature.* 2015;518:197–206.

17. Shungin D, Winkler TW, Croteau-Chonka DC, Ferreira T, Locke AE, Mägi R, Strawbridge RJ, Pers TH, Fischer K, Justice AE,.et al. New genetic loci link adipose and insulin biology to body fat distribution. *Nature.* 2015;518:187–196.

18. Thorleifsson G, Walters GB, Gudbjartsson DF, Steinthorsdottir V, Sulem P, Helgadottir A, Styrkarsdottir U, Gretarsdottir S, Thorlacius S, Jonsdottir I, et al. Genome-wide association yields new sequence variants at seven loci that associate with measures of obesity. *Nat Genet.* 2009;41:18–24.

19. Ehret GB, Munroe PB, Rice KM, Bochud M, Johnson AD, Chasman DI, Smith AV, Tobin MD, Verwoert GC, Hwang SJ, et al. Genetic variants in novel pathways influence blood pressure and cardiovascular disease risk. *Nature.* 2011;478:103–109.

20. Willer CJ, Sanna S, Jackson AU, Scuteri A, Bonnycastle LL, Clarke R, Heath SC, Timpson NJ, Najjar SS, Stringham HM, et al. Newly identified loci that influence lipid concentrations and risk of coronary artery disease. *Nat Genet*. 2008; 40:161–169.

21. Barrett JC, Clayton DG, Concannon P, Akolkar B, Cooper JD, Erlich HA, Julier C, Morahan G, Nerup J, Nierras C, et al. Genome-wide association study and meta-analysis find that over 40 loci affect risk of type 1 diabetes. *Nat Genet.* 2009;41:703–707.

American Heart Association.
Circulation: Genomic and Precision Medicine

22. Saxena R, Hivert M-F, Langenberg C, Tanaka T, Pankow JS, Vollenweider P, Lyssenko V, Bouatia-Naji N, Dupuis J, Jackson AU, et al. Genetic variation in *GIPR* influences the glucose and insulin responses to an oral glucose challenge. *Nat Genet.* 2010;42:142–148.

23. Manning AK, Hivert M-F, Scott RA, Grimsby JL, Bouatia-Naji N, Chen H, Rybin D, Liu CT, Bielak LF, Prokopenko I, et al. A genome-wide approach accounting for body mass index identifies genetic variants influencing fasting glycemic traits and insulin resistance. *Nat Genet.* 2012;44:659–669.

24. Dupuis J, Langenberg C, Prokopenko I, Saxena R, Soranzo N, Jackson AU, Johnson T, Kanoni S, Ladenvall C, Lagou V, et al. New genetic loci implicated in fasting glucose homeostasis and their impact on type 2 diabetes risk. *Nat Genet.* 2010;42:105–116.

25. Soranzo N, Sanna S, Wheeler E, Gieger C, Radke D, Dupuis J, et al. Common variants at 10 genomic loci influence hemoglobin A1C levels via glycemic and nonglycemic pathways. *Diabetes.* 2010;59:3229–3239.

26. Strawbridge RJ, Dupuis J, Prokopenko I, Barker A, Ahlqvist E, Rybin D, Petrie JR, Travers ME, Bouatia-Naji N, Dimas AS, et al. Genome-wide association identifies nine common variants associated with fasting proinsulin levels and provides new insights into the pathophysiology of type 2 diabetes. *Diabetes.* 2011;60:2624–2634.

27. Lotta LA, Gulati P, Day FR, Payne F, Ongen H, van de Bunt M, Gaulton KJ, Eicher JD, Sharp SJ, Luan J, et al. Integrative genomic analysis implicates limited peripheral adipose storage capacity in the pathogenesis of human insulin resistance. *Nat Genet.* 2017;49:17–26.

28. Scott RA, Lagou V, Welch RP, Wheeler E, Montasser ME, Luan J, Mägi R, Strawbridge RJ, Rehnberg E, Gustafsson S, et al. Large-scale association analyses identify new loci influencing glycemic traits and provide insight into the underlying biological pathways. *Nat Genet*. 2012;44:991–1005.

29. van Leeuwen EM, Smouter FAS, Kam-Thong T, Karbalai N, Smith A V., Harris TB, Launer LJ, Sitlani CM, Li G, et al. The challenges of genome-wide interaction studies: Lessons to learn from the analysis of HDL blood levels. *PLoS One.* 2014;9:e109290.

30. Huang Y, Cai X, Mai W, Li M, Hu Y. Association between prediabetes and risk of cardiovascular disease and all-cause mortality: Systematic review and meta-analysis. *BMJ.* 2016;355:i5953.

31. Pirola L, Balcerczyk A, Tothill RW, Haviv I, Kaspi A, Lunke S, Ziemann M, Karagiannis T, Tonna S, Kowalczyk A, et al. Genome-wide analysis distinguishes hyperglycemia regulated epigenetic signatures of primary vascular cells. *Genome Res.* 2011;21:1601–1615.

32. Strawbridge, R J & van Zuydam NR. Shared genetic contribution of type 2 diabetes and cardiovascular disease: implications for prognosis and treatment. *Curr Diab Rep*. 2018:18:59

Circulation: Genomic and Precision Medicine

Figure Legends:

Figure 1: Study Design. In the discovery meta-analyses, we performed four different meta-analyses of coronary artery disease (CAD): in all individuals irrespective of diabetes status; in all individuals corrected for diabetes stats; and stratified by diabetes status. We examined allelic effects within strata to identify stratum specific CAD associated variants, and between strata to identify variants that may interact with diabetes status to modify the risk of CAD. We selected variants that achieved p value$<1\times10^{-4}$ for association with CAD in at least one of the following analyses: all individuals combined regardless of T2D status; subjects with T2D only; subjects without diabetes; or the interaction analysis. The replication analysis was performed in independent samples using the same study design as the discovery analysis.

American Heart Association.
Circulation: Genomic and Precision Medicine

Figure 2: Manhattan and QQ plots from: (A) a meta-analysis that combined allelic effects on coronary artery disease (CAD) from subjects with type 2 diabetes (T2D) and without diabetes; and (B) corrected for T2D status to identify variants associated with CAD irrespective of T2D status; (C) a meta-analysis of allelic effects on CAD in subjects with T2D to identify loci that may influence the development of CAD in the context of T2D; (D) a meta-analysis of allelic effects on CAD in the absence of diabetes to identify loci that may influence the development of CAD in the absence of diabetes; and (E) an interaction analysis to identify loci that may interact with T2D to modify the risk of CAD. The effective sample size was based on the combined discovery and replication sample of 184, 250 subjects.

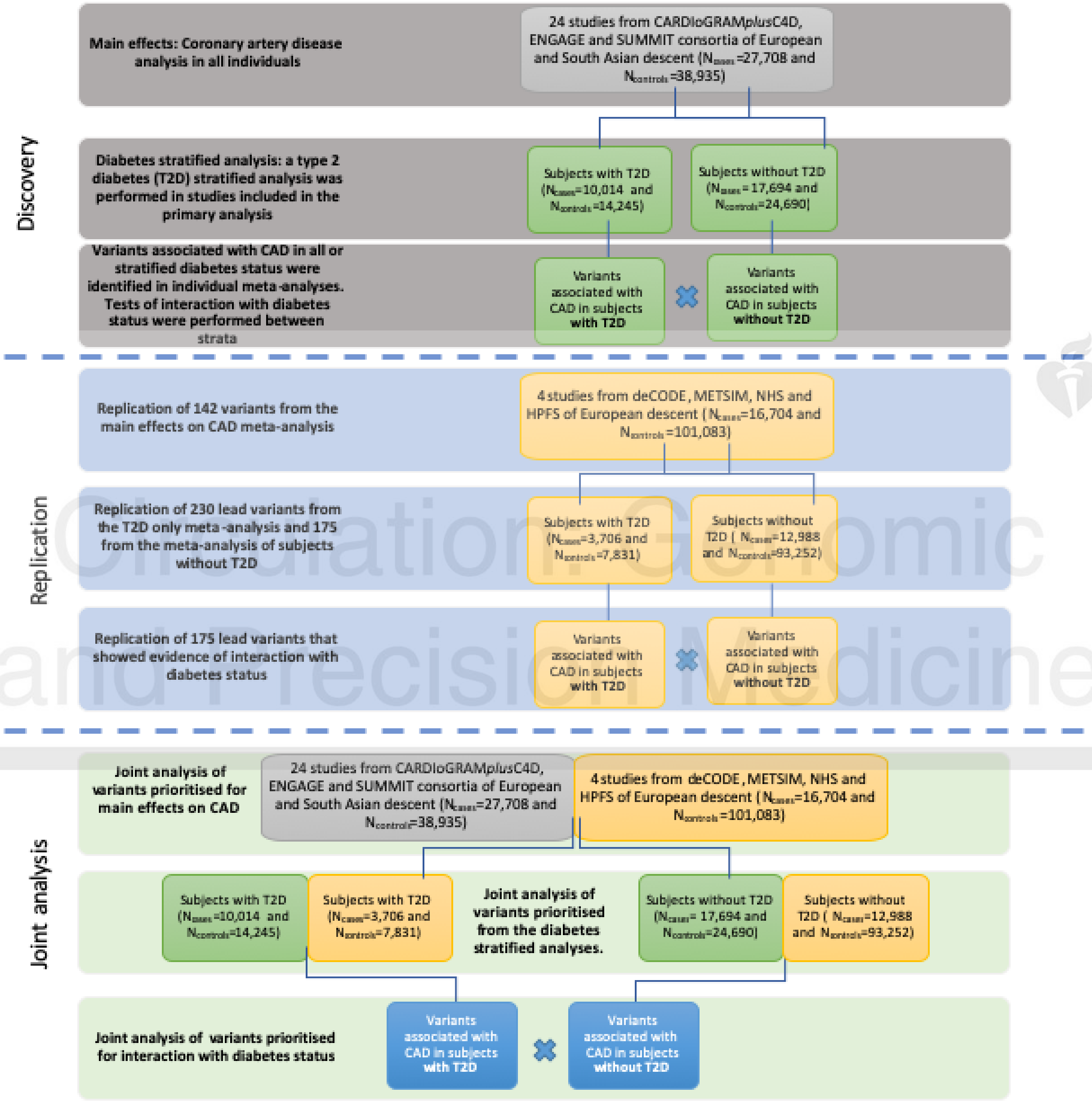

Circulation: Genomic and Precision Medicine
American Heart Association.

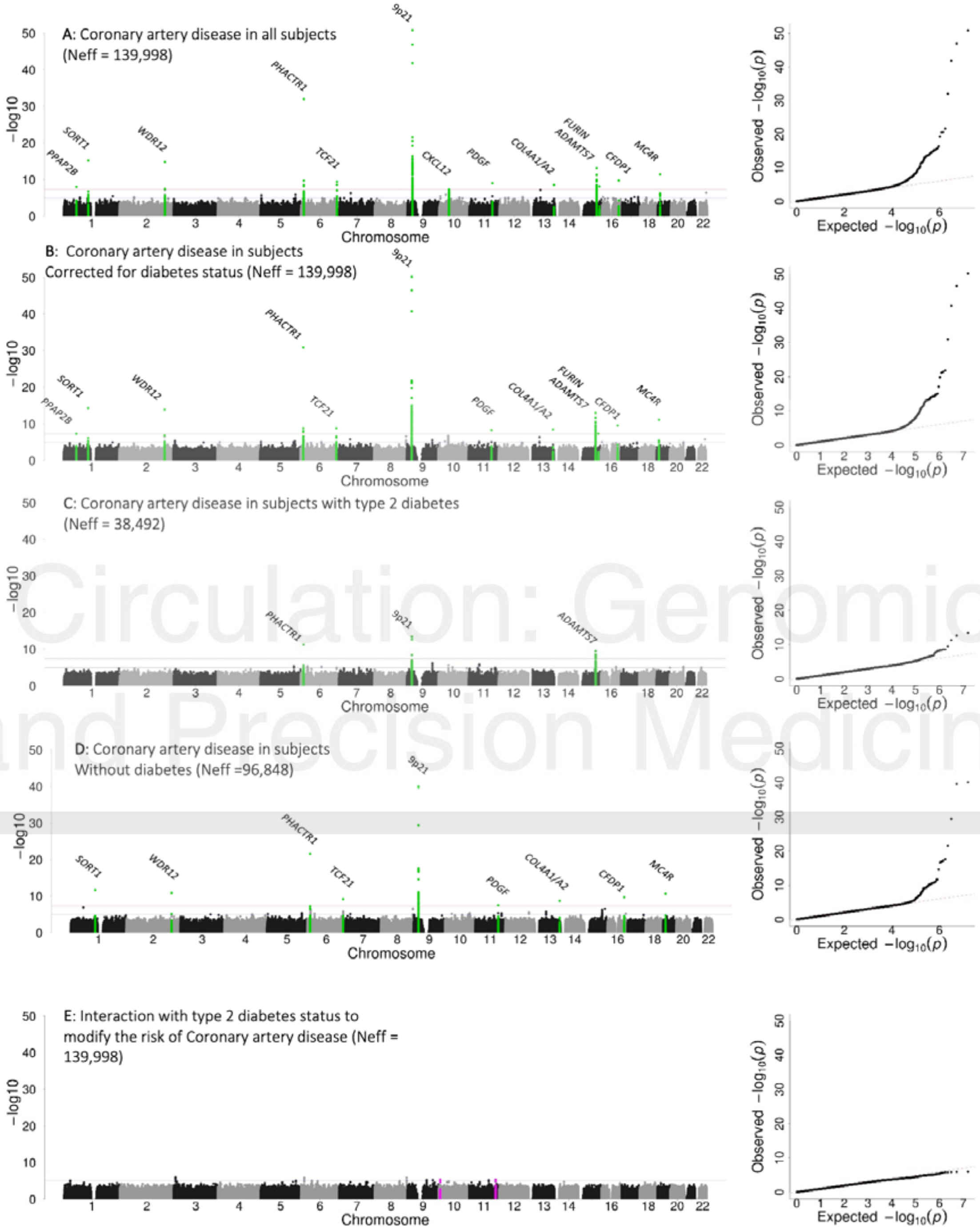

Circulation: Genomic and Precision Medicine

American Heart Association.
